# Cross-platform comparison of immune signatures in immunotherapy-treated patients with advanced melanoma using a rank-based scoring approach

**DOI:** 10.1186/s12967-023-04092-9

**Published:** 2023-04-13

**Authors:** Yizhe Mao, Tuba N. Gide, Nurudeen A. Adegoke, Camelia Quek, Nigel Maher, Alison Potter, Ellis Patrick, Robyn P. M. Saw, John F. Thompson, Andrew J. Spillane, Kerwin F. Shannon, Matteo S. Carlino, Serigne N. Lo, Alexander M. Menzies, Inês Pires da Silva, Georgina V. Long, Richard A. Scolyer, James S. Wilmott

**Affiliations:** 1 Melanoma Institute Australia, The University of Sydney, Sydney, NSW Australia; 2 Faculty of Medicine and Health, The University of Sydney, Sydney, NSW Australia; 3 Charles Perkins Centre, The University of Sydney, Sydney, NSW Australia; 4 Royal Prince Alfred Hospital and NSW Health Pathology, Sydney, NSW Australia; 5 School of Mathematics and Statistics, The University of Sydney, Sydney, NSW Australia; 6 The Westmead Institute for Medical Research, The University of Sydney, Westmead, NSW Australia; 7 Royal Prince Alfred Hospital, Camperdown, NSW Australia; 8 Mater Hospital, North Sydney, Sydney, NSW Australia; 9 Royal North Shore Hospital, Sydney, Australia; 10 Chris O’Brien Lifehouse, Camperdown, NSW Australia; 11 Westmead and Blacktown Hospitals, Sydney, NSW Australia

**Keywords:** Advanced melanoma, Immunotherapy, Immune signature, Gene expression profile, Single-sample signature score, Cross-platform analyses

## Abstract

**Background:**

Gene expression profiling is increasingly being utilised as a diagnostic, prognostic and predictive tool for managing cancer patients. Single-sample scoring approach has been developed to alleviate instability of signature scores due to variations from sample composition. However, it is a challenge to achieve comparable signature scores across different expressional platforms.

**Methods:**

The pre-treatment biopsies from a total of 158 patients, who have received single-agent anti-PD-1 (n = 84) or anti-PD-1 + anti-CTLA-4 therapy (n = 74), were performed using NanoString PanCancer IO360 Panel. Multiple immune-related signature scores were measured from a single-sample rank-based scoring approach, *singscore*. We assessed the reproducibility and the performance in reporting immune profile of *singscore* based on NanoString assay in advance melanoma. To conduct cross-platform analyses, *singscores* between the immune profiles of NanoString assay and the previous orthogonal whole transcriptome sequencing (WTS) data were compared through linear regression and cross-platform prediction.

**Results:**

*singscore*-derived signature scores reported significantly high scores in responders in multiple PD-1, MHC-1-, CD8 T-cell-, antigen presentation-, cytokine- and chemokine-related signatures. We found that *singscore* provided stable and reproducible signature scores among the repeats in different batches and cross-sample normalisations. The cross-platform comparisons confirmed that *singscores* derived via NanoString and WTS were comparable. When *singscore* of WTS generated by the overlapping genes to the NanoString gene set, the signatures generated highly correlated cross-platform scores (Spearman correlation interquartile range (IQR) [0.88, 0.92] and *r*^*2*^ IQR [0.77, 0.81]) and better prediction on cross-platform response (AUC = 86.3%). The model suggested that Tumour Inflammation Signature (TIS) and Personalised Immunotherapy Platform (PIP) PD-1 are informative signatures for predicting immunotherapy-response outcomes in advanced melanoma patients treated with anti-PD-1-based therapies.

**Conclusions:**

Overall, the outcome of this study confirms that *singscore* based on NanoString data is a feasible approach to produce reliable signature scores for determining patients’ immune profiles and the potential clinical utility in biomarker implementation, as well as to conduct cross-platform comparisons, such as WTS.

**Supplementary Information:**

The online version contains supplementary material available at 10.1186/s12967-023-04092-9.

## Background

Gene expression profiling is commonly used to investigate the immune profiles of the tumour microenvironment for cancer patients, particularly in the setting of response and survival predictions for cancer patients treated with anti-PD-1 monotherapy and anti-PD-1 + anti-CTLA-4 therapy [1–4]. Multiple methods are used to generate the raw gene expression data, including whole transcriptome sequencing (WTS), which is a comprehensive and powerful tool used to identify a wide spectrum of immune-related gene expression profiles, but often requires significant infrastructure and resource cost. Alternatively, targeted panel-based approaches, such as the NanoString nCounter^®^ platform, are rapid and scalable to assess the immune profile in the tumour microenvironment. Other studies have demonstrated the gene expression patterns by NanoString nCounter^®^ PanCancer IO 360^™^ are highly correlated to other platforms, including WTS and HTG EdgeSeq [5, 6]. For data analysis, NanoString nCounter® has an in-built analysis tool, nSolver^™^ Version 4.0, which provides extensive end-to-end solutions for researchers including biologists with no prior bioinformatic experience to perform quality control (QC), normalisation and downstream analysis, including differential expression, gene set analysis and pathway scoring [7, 8].

Gene set scoring analysis provides inter-sample insights on variations and concordances of the transcriptome. An issue for stably generating single sample signature scores is that they are commonly affected by the number of samples and normalisation of expression data across an entire cohort. One method, gene set variation analysis (GSVA), utilises a kernel function to estimate the gene expression distribution across the samples in a cohort [9], and single sample gene set enrichment analysis (ssGSEA) normalises the final scores across samples to achieve comparable results [10, 11]. The reproducibility of signature score via these methods may be critical, since the scores are impacted if the number of samples or genes is changed. The *singscore* [11] method is a rank-based scoring approach that evaluates the absolute average deviation of a gene from the median rank in a gene list. It provides a simple, stable, and faster scoring approach, even in the single sample scale, compared to other signature scoring methods, including GSVA, ssGSEA, PLAGE and combination z-scores [11].

Our previous study reported the immune profiles of melanoma patients treated with anti-PD-1 monotherapy or combined anti-CTLA-4 dual therapy based on WTS data [3]. This study investigated the gene expression profiles of additional metastatic melanoma patients generated on the NanoString nCounter® PanCancer IO 360™ platform. We evaluated the reproducibility and clinical significance of signature scores for immunotherapy response status on the NanoString platform by the rank-based scoring method, *singscore*. We then identify an integration method that enables concordant *singscores* to be derived via targeted gene expression platforms (Nanostring) or WTS from the same sample to enable use of datasets from generated via different platforms.

## Methods

### Patient cohort

Patients with advanced melanoma were treated with standard-of-care single agent anti-PD-1 (nivolumab or pembrolizumab) or a combined anti-PD-1 + anti-CTLA-4 (ipilimumab) therapy. Patients were retrospectively identified, based on formalin-fixed paraffin-embedded (FFPE) tissue availability. Patients were excluded from the study if they lacked an available baseline pre-treatment biopsy, or the biopsy had less than 100 melanoma cells following pathological review (AJP/NM). Patient response was determined using the RECIST 1.1 criteria [12]. Responders were categorised as patients with a RECIST response of complete response (CR), partial response (PR), or stable disease (SD) greater than 6 months with no progression, while non-responders were categorised as progressive disease (PD) or SD for less than or equal to 6 months before disease progression.

### RNA isolation and NanoString profiling

Total RNA was isolated from macro-dissected FFPE tissue sections using the AllPrep DNA/RNA FFPE Kit (Qiagen) or High Pure FFPET RNA Isolation Kit (Roche) according to the manufacturer’s instructions. RNA quantity was assessed on Qubit, and RNA integrity was assessed using the Tapestation system (Agilent). Total RNA samples (60 ng/ul, total 200 ng) were used as input for the NanoString PanCancer IO360 Panel, run on the nCounter MAX/FLEX prep station and scanner. Samples were hybridised for 20 h, and each cartridge contained a panel standard.

### NanoString expression data

Data importing, normalisation, and sample calibration were conducted on the RCC files in nSolver^™^ Version 4.0 [7]. The 185 samples’ files were imported with QC in the default setting. Next, in the MultiRLF analysis, background thresholding, and positive control normalisation followed the default setting. The CodeSet Content normalisation was applied against 19 housekeeping genes (HKGs), where the gene, *STK11IP,* was excluded due to a higher mean standard deviation (%CV) value than the others. The Panel Standard in each cartridge was selected in the CodeSet Calibration step. The samples with overall low expression (HKG normalisation ratio ≥ 10) than the others were flagged (Additional file 1: Fig. S1A, B). Finally, the normalised NanoString expression table filtered out samples with *mRNA positive Normalisation Flag* or *mRNA Content Normalisation Flag*. There were 165 samples with 770 genes in normalised NanoString count data.

### WTS expression data

Prior WTS normalised count data on overlapping patients (35 samples) was downloaded [13] (Additional file 2: Table S2). The WTS table contained 22,300 genes across all samples. To match the NanoString Probe names, six genes in WTS were merged into three genes; ten genes were replaced by their aliases’ names; and eight NanoString Probes were filtered out as there was no matched gene in the WTS gene list (Additional file 2: Table S4).

### Calculation of signature scores by *singscore*

A curated set of 81 signatures was used in this study based on their known utility in the context of immunotherapy response [14–21], internal NanoString signatures, msigdb [22, 23] and signatures derived in this study based on the receptor and ligand pairing of immunotherapeutic agents in clinical trials [24] (Additional file 2: Table S5). The scoring system utilised the R (version 4.2.0) package *singscore* (1.16.0) [11]. Only 63 of 81 signatures which contained all genes within the signature in the overlapping 762 genes were applied when calculating *singscores* for the WTS data. All *singscores* were calculated in the undirected gene signatures mode. *Singscore* allows introducing stable genes to calibrate ranks across samples from different transcriptomic data [25]. When introducing a list of “*n*” stable genes, the rank of genes in each sample was stratified into “*n*” levels based on the location of these stable genes.

All rankings were conducted by *rankGenes()* function in *singscore*. It returned the per sample gene ranks assigned with integers from 0 in an ascending order based on the order of count values. The genes with the same count value were assigned the identical rank.

**Fig. 1:**
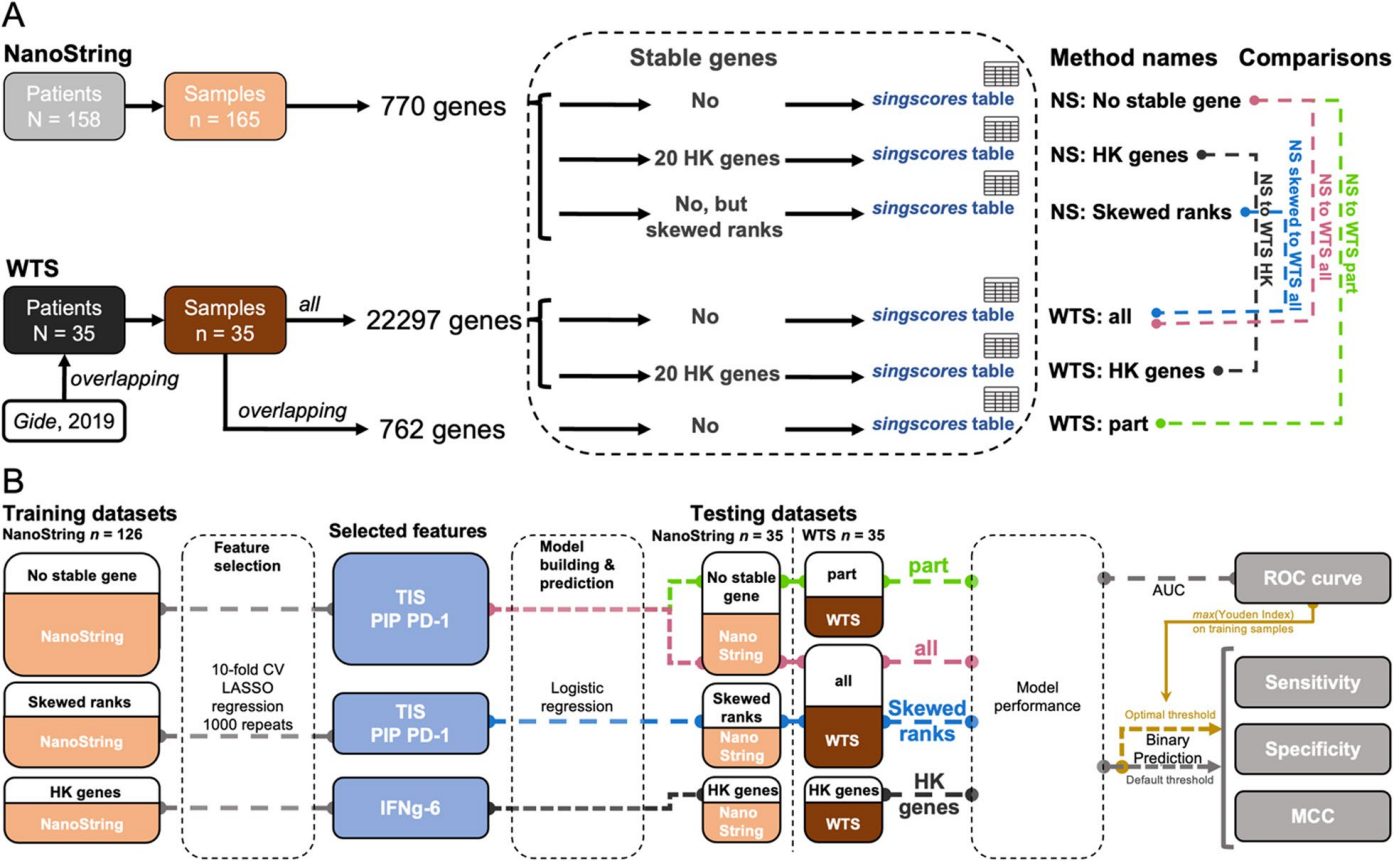
Workflow outlining *singscores* calculation across all samples and cross-platform predictive model building. **A** The workflow displays several methods to calculate *singscores* based on different ranking strategies. Both platforms applied 20 genes labelled as HKG in NanoString probes for calibration, named the “HK genes” methods. Without introducing any stable gene, in the NanoString platform *singscore* directly used such ranks in the “No stable gene” method and used the “Skewed ranks” method based on the regression (Additional file 1: Fig. S7E). The “all” and “part” methods in the WTS platform also did not include any stable gene, but “all” used all genes to rank, and “part” used overlapping genes to rank. “NS” indicates data from the NanoString assay; “WTS” indicates data from the overlapping whole transcriptome Sequencing samples. Four different coloured dot-dashed lines represent four pairs of cross-platform comparisons. **B** The workflow displays the processes of evaluating multiple cross-platform predictions by signatures’ *singscores*. The feature selection by tenfold CV LASSO regression and model building based on the three *singscore* tables derived from three singscore-calculating approaches in **A** from 126 NanoString samples. Two types of testing datasets were based on 35 overlapping samples from NanoString and WTS platforms. AUC, sensitivity and specificity and MCC were applied to evaluate model performance

For NanoString data, the rankings were based on three options (Fig. 1A): (1) “No stable gene”: without any stable gene, (2) “HK genes”: 20 NanoString in-built HKGs as stable genes, and (3) “Skewed ranks”: the rank of genes from the “No stable gene” method was skewed by the coefficients from a linear regression (Additional file 1: Fig. S7E). This regression was generated by fitting a uniform distribution against the median ranks of the overlapping 762 genes in the WTS platform. The assumption was that the rank of genes in the NanoString platform follows a uniform distribution.

For WTS data, two types of WTS gene lists were applied in this study: all 22,297 genes and overlapping 762 genes. Therefore, the *rankGenes()* function had three ranking options (Fig. 1A): (1) “all”: without any stable gene, on all 22297 genes, (2) “part”: without any stable gene, on all 762 overlapping genes, and (3) “HK genes”: 20 NanoString in-built HKGs as stable genes.

### Consistency of gene ranks

To evaluate the stability of ranks in a list of genes, gene rank consistency between NanoString and WTS data was measured, which was referred to the process of gene-wise rank consistency measurement [25]. For a list of genes, the gene-wise rank consistency of each gene was the average value of pairwise consistency score. The pairwise consistency score for one gene was computed by calculating the preservation of order on this gene to others. In each pair, order preservation was defined as the percentage of samples in NanoString data preserving the same orders to reference order. The reference order was based on the order of the median gene ranks in WTS data. Such average consistency scores were measured on 20 HKGs to evaluate cross-platform rank consistency, and 762 overlapping genes to find potential platform-specific “stable genes”.

### Identifying differences in responder status

To define the significant difference between response status, the *p*-values from a statistical test were conducted through a multiple testing correction process, *Benjamini-Hochberg* (*BH*) adjustment, to avoid type 1 errors where False Discovery Ratio (FDR) ≤ 0.05 was used as the significant threshold. For *singscore* differences, the significantly different signatures between responders and non-responders were identified using the *Mann Whitney* Wilcoxon test. The NanoString in-built differential expression analysis and pathway scoring were conducted by Advanced analysis in nSolver 4.0 [8] on 165 no normalisation-flagged samples. The “Custom Analysis” was applied. The “Experiment Type” was set as “*MultiRLF Merge (standard experiments merged)*”. On the “Differential Expression” option page, the optimal option was chosen, and the *p*-value was adjusted using the *BH* procedure with a threshold of 0.05. The pathway scoring followed the default setting. These pathway scoring results were further measured on *Mann Whitney* Wilcoxon test to measure the significant differences between response status.

### Similarity comparisons

Spearman correlation (*r*) and linear regression were applied to evaluate similarity for any pair of comparison.

In repeated comparisons on NanoString data, 12 repeated samples from 5 patients formed 9 pairs of comparisons. Linear regression was conducted on raw and normalised counts and *singscore* values (from the “No stable gene” method) within each pair. In *singscore* stability comparisons of NanoString data, per sample linear regression was run between *singscores* based on the raw and normalised count data.

In cross-platform comparisons, the 35 overlapping samples’ *singscores* generated by different methods from the NanoString platform were fitted against the *singscores* generated by different WTS platform methods. There were four types of comparisons: (1) “NS to WTS HK”: *singscores* from utilizing the housekeeping gene “HK genes” method in NanoString to the scores from the “HK genes” method in WTS, (2–3) “NS to WTS all” and “NS to WTS part”: *singscores* from the “No stable gene” method in NanoString to the scores from all genes “all” and subset overlapping genes “part” methods in WTS, and (4) “NS skewed to WTS all”: *singscores* from the “Skewed ranks” method in NanoString to the scores from the “all” method in WTS (Fig. 1A).

### Cross-platforms predictions

The prediction models were built on *singscores* in the NanoString platform to assess the utility of each signature quantification approach. Thirty-nine (35 overlapping samples with 4 relative repeats) out of 165 samples were excluded from the NanoString. The remaining 126 non-overlapping samples in NanoString data were used to predict the 35 overlapping samples. There were two testing datasets: (1) 35 overlapping samples from the WTS platform and (2) from the NanoString platform. The three different *singscore* tables from NanoString platform were divided into three separated training and NanoString testing datasets. The three different *singscore* tables from WTS platform were used as WTS testing datasets. The four pairs of cross-platform predictions are same to the cross-platform comparisons in Fig. 1A.

The tenfold cross-validation using LASSO regression was used for feature selection. The feature selection kept non-zero coefficients under *λ* value, which provided the largest mean Area Under the Curve (AUC) value on the ROC curve in tenfold cross-validation based on the training dataset. To achieve more robust and reproducible features, this feature selection process was repeated 1000 times and the frequency of selected features was recorded. The predictive models were built by the logistic regression with the binary cluster using the frequently selected features. The sensitivity, specificity and Matthews Correlation Coefficient (MCC) values of the predicted binary outcomes (responder/non-responder), as well as AUC of predictions were used to evaluate the predictive performance of the model. There were two types of thresholds (probability of responding to PD-1-based immunotherapies) applied in binary classification of the predicted response status. The one was 0.5 (default). The other was based on the optimal threshold which provided the maximum Youden index in the predictive ROC curve of the training dataset in each model (Fig. 1B).

## Results

### Patients characteristics

A greater number of patients were classified as responders (n = 90) compared to non-responders (n = 68). Anti-PD-1 + anti-CTLA-4 treated patients achieved a higher response rate (47 out of 74, 64%) than those treated with anti-PD-1 monotherapy (43 out of 84, 51%), but lack statistical significance. Forty-six percent of the samples were subcutaneous specimens (73/158) and 28% were lymph node specimens (44/158). No significant association was found between the patients’ responses and site of biopsy (Table 1).

**Table 1:** Clinical characteristics of patients in Nanostring cohort after QC and normalisation

Characteristic		All (N = 158)	Responder (N = 90)	Non-responder (N = 68)	P-value
Patients with repeats, *N*		5	3	2	
	Additional repeats, No	7	4	3	
Treatment, *N*. (%):	IPI + PD1^c^	74 (46.8)	47 (52.2)	27 (39.7)	0.1615^a^
	PD1^c^	84 (53.2)	43 (47.8)	41 (60.3)	
Biopsy sites, *N*. (%):					0.1461^b^
	Brain	15 (9.5)	9 (10)	6 (8.8)	
	Liver	2 (1.3)	2 (2.2)		
	Lung	7 (4.4)	4 (4.4)	3 (4.4)	
	Lymph node	44 (27.8)	21 (23.3)	23 (33.8)	
	Mucosa	2 (1.3)		2 (2.9)	
	Primary	6 (3.8)	5 (5.6)	1 (1.5)	
	Small bowel	3 (1.9)	2 (2.2)	1 (1.5)	
	Subcutaneous	73 (46.2)	41 (45.6)	32 (47.1)	
	Other	6 (3.8)	6 (6.7)		

*N* indicates number

^a^Pearson’s *Chi-squared* test with Yates’ continuity correction

^b^Pearson’s *Chi-squared* test

^c^IPI: ipilimumab; PD1: nivolumab or pembrolizumab

Samples were assessed for batch variability pre-normalisation using raw counts. Samples from Cartridge 13–17 (batch 3) displayed an overall lower expression than the other cartridges (Additional file 1: Fig. S2A). There was no clear separation in PCA plots for response status, type of treatments, and site of biopsy (Additional file 1: Fig. S3B-D). Following normalisation, the samples had relatively similar dual-peak distributions (Additional file 1: Fig. S1D) but varied in quantiles (Additional file 1: Fig. S1C). No clear separation was observed in response status or type of immunotherapy (Additional file 1: Fig. S2B). However, the batch effect was alleviated following normalisation (Additional file 1: Fig. S3A, E). All HKGs’ Relative Standard Deviations (RSDs) shrank after normalisation in NanoString data (Additional file 1: Fig. S6A, C). All WTS samples had similar distributions and quantiles, but these were normalised counts (Additional file 1: Fig. S1E, F).

### Differences in gene expression profiles between responders and non-responders

**Fig. 2:**
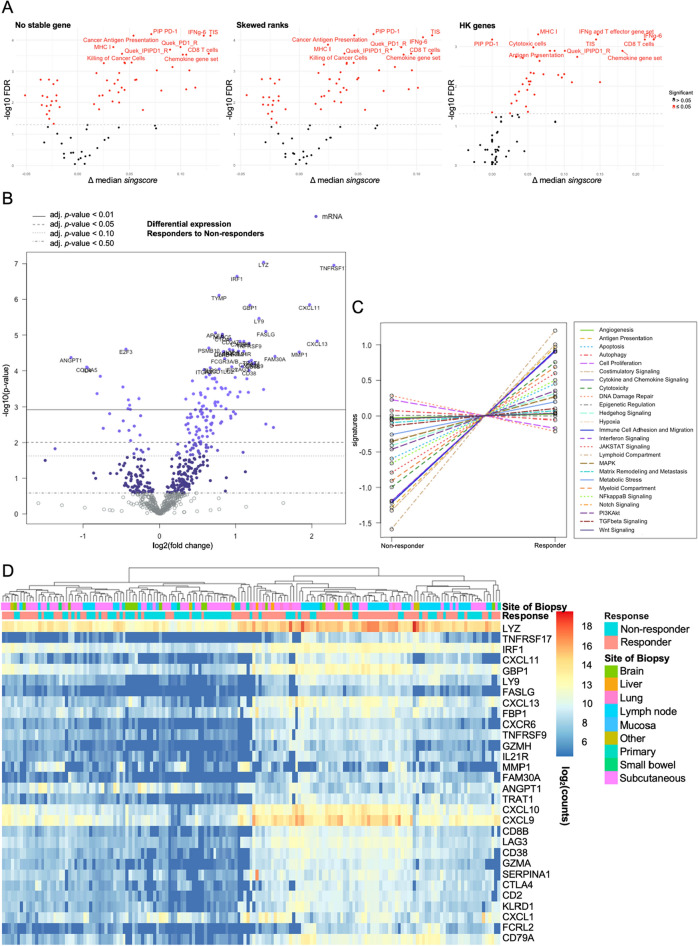

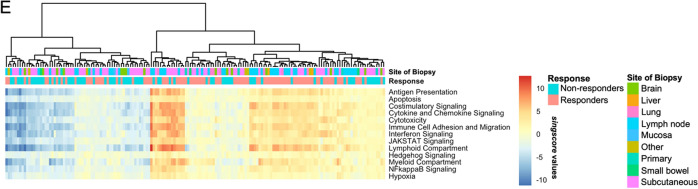
Differences in response status by NanoString data. **A** Volcano plot of *singscores* based on the three *singscore*-calculating approaches. The red dots denote gene expression signatures (FDR ≤ 0.05) detailing the top 10 significant signatures. The y-axis is –log_10_-transformed FDR. The x-axis is the difference in the median *singscores* between responders and non-responders. **B** Volcano plot of gene expressions from Advanced Analysis in nSolver4.0. The y-axis is –log_10_-transformed adjusted *p*-values. The x-axis is log_2_-transformed fold change. **C** The plots based on scores of 25 in-built pathways from Advanced Analysis in nSolver4.0. Each dot represents the centralized average scores in each signature in responders/non-responders. **D** Heatmap of 30 DEGs with adj. *p*-values ≤ 0.05 and |log_2_FC|≥ 1. The color shows the log_2_(count). **E** Heatmaps of scores of 13 significant in-built pathways with hierarchical clustering. The color indicates the pathway score

We first compared results obtained from NanoString assay using *singscore* analysis processes to evaluate the differences in gene expression signatures in association with responders and non-responders. We processed the *singscores* without applying stable gene normalisation (No stable gene), with HKGs normalisation (HK genes), using the skewed ranks method (Skewed ranks) and then tested for significant (FDR ≤ 0.05) differences in responding and non-responding patients. Of the 81 immune-related signatures (Additional file 2: Table S5), the same list of 57 signatures in the “No stable gene” and “Skewed ranks” methods (Additional file 3: Table S6-7), 39 signatures in the “HK genes” method (Additional file 3: Table S8) passed the threshold. The signatures, including Personalised Immunotherapy Platform (PIP) PD-1, Tumour Inflammation Signature (TIS) and CD8 T cells are the top three significant signatures (adj. *p*-value ≤ 2 × 10^–4^ and difference of median *singscores* ≥ 0.1) where responders display high *singscores* in both “No stable gene” and “Skewed ranks” *singscore*-calculating approaches (Fig. 2A, Additional file 3: Fig. S6, S8). The significant signatures based on the “HK genes” *singscore*-calculating approach provided lower signature scores (Additional file 1: Fig. S4), but larger differences in median *singscores* (Fig. 2A). Regardless of which *singscore*-calculating approach was used, the responding patients always showed significantly higher *singscores* in multiple CD8 T cell-, IFN-gamma(g)-, PD-1-, MHC-I-, and cytotoxic-related signatures (Additional file 3: Table S6–S8). Additionally, the “No stable gene” and “Skewed ranks” methods identified signatures relating to autophagy, hypoxia, angiogenesis, DNA damage repair, cell proliferation, as well as multiple cancer-related signalling pathways including PI3K/Akt, Notch, TGFβ, MAPK, and WNT, which had significantly higher *singscores* in non-responders (Additional file 3: Table S6, S8). However, none of *singscore*-calculating approach can provide a good binary classification of response status based on the significant signatures using hierarchal clustering (Additional file 1: Fig. S4). Furthermore, lymph node specimens (61.4%: 27 out of 48 are in the left cluster in Additional file 1: Fig. S4A) displayed higher scores for multiple immune cell-related signatures, including CD8 T cells, Exhausted CD8, T cells, CD45, and B cells, compared to other specimens. The Wilcoxon test also showed the statistical significance of higher scores in these signatures in the samples extracted from lymph node samples (Additional file 3: Table S9).

We then sought to analyse the same data using the nSolver4.0 Advanced Analysis pipeline. Differential expression analysis between the response groups identified 142 significant differential expression genes (adj. *p*-value ≤ 0.05), of which 30 had a log_2_-fold change (FC) larger or equal to 1. There were more significantly highly expressed genes in responders (29 genes log_2_FC ≥ 1: *TNFRSF17*, *LYZ*, *IRF1*, *CXCL11*, *GBP1*, *LY9*, *FASLG*, *CXCL13*, *FBP1*, *CXCR6*, *TNFRSF9*, *MMP1*, *IL21R*, *GZMH*, *FAM30A*, *TRAT1*, *CXCL10*, *CXCL9*, *CD8B*, *LAG3*, *CD38*, *GZMA*, *SERPINA1*, *CD2*, *CTLA4*, *KLRD1*, *CXCL1*, *FCRL2*, *CD79A*) than in non-responders (1 gene log_2_FC ≤ -1: *ANGPT1*) (Fig. 2B, Additional file 4: Table S10). These 30 DEGs classified samples into to two clusters, where 47 out of 71 (66.2%) non-responders are in the left cluster and 58 out of 94 (61.7%) responders are in the right cluster (Fig. 2D). Based on 25 NanoString in-built pathway scores, Antigen Presentation, Apoptosis, and Costimulatory Signalling were the top three signatures with significantly higher median scores (adj. *p*-value ≤ 4 × 10^–3^) in the responders, and DNA Damage Repair and Cell Proliferation displayed higher median scores in the non-responders, but these did not reach significance (Fig. 2C, Additional file 4: Table S12). The top two predominant clusters are 37 out of 71 (52.1%) non-responders in the left cluster and 68 out of 94 (72.3%) responders in the right cluster (Fig. 2E).

### Stability and reproducibility of NanoString and *singscore*

**Fig. 3:**
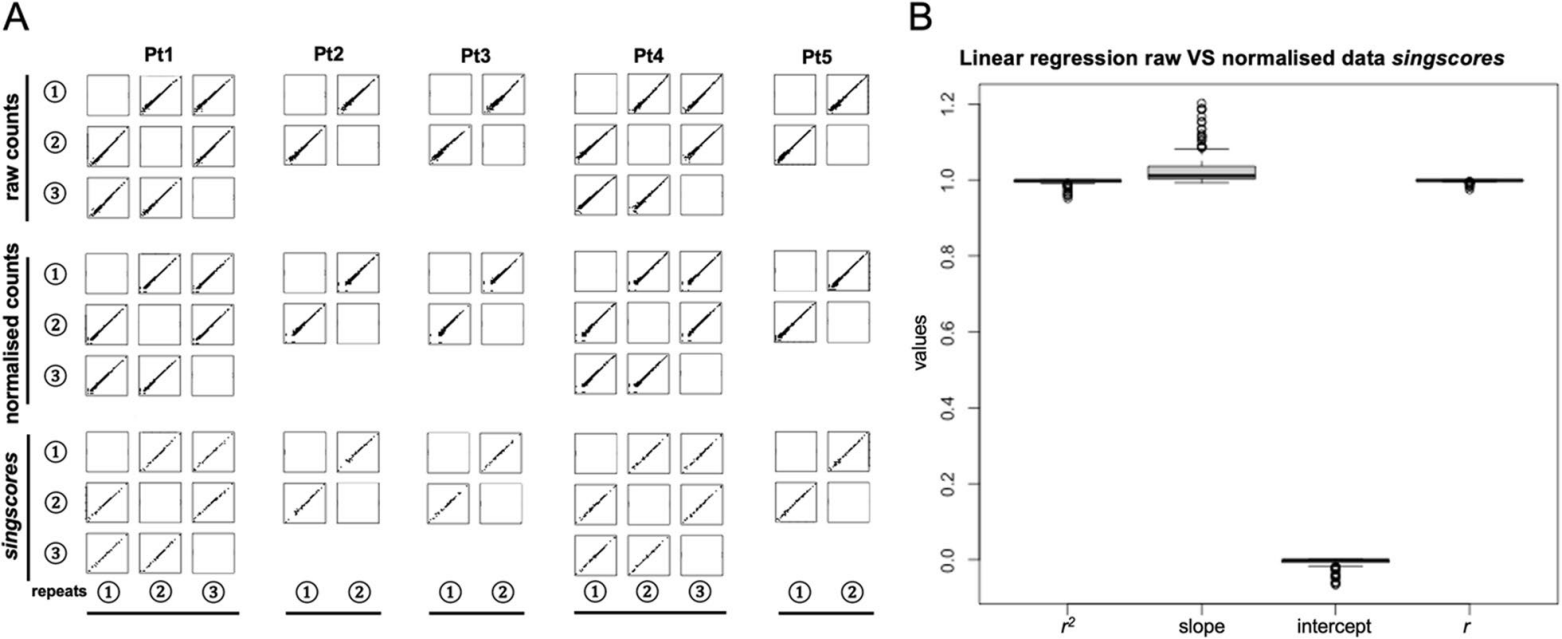
Comparisons of counts and *singscores* within samples from NanoString assay. **A** Linear regression between 12 NanoString repeats from 5 samples. The repeats were derived from samples from the same patients (Pt1-5). Linear regression of raw count data (top lane); normalised count data (middle lane); *singscores* using the “No stable gene” method (bottom lane). **B** Boxplot of Spearman correlation (*r*) and Linear regression coefficients, including the *r*^*2*^, slope and intercept, between *singscores* of 81 signatures derived from raw and normalised NanoString counts using “No stable gene” method of 165 samples

Here, we aimed to identify a robust analysis process that could provide reproducible and clinically relevant gene expression signature scores. We therefore tested the stability and reliability of *singscores* across repeats among different processing batches and between the scores derived from raw and normalised count data in the same sample. Each pair of repeats displayed high similarity in raw counts, normalised counts and *singscores*. When comparing the normalised counts, few gene variations could be observed in the low count region (Additional file 1: Fig. S5). Correlations and linear regression *r*^*2*^ were larger than 0.99 in all pairs of repeats (Fig. 3A). Highly consistent signature scores can be observed in the *singscores* from raw and normalised counts. All 165 samples displayed correlations, *r*^*2*^ and slopes close to 1 (interquartile range (IQR) [0.998, 0.999], [0.995, 0.999], and [1.003,1.036]), and intercepts close to 0 (IQR [− 0.007, 0]) (Fig. 3B).

### Cross-platform gene ranks consistencies

**Fig. 4:**
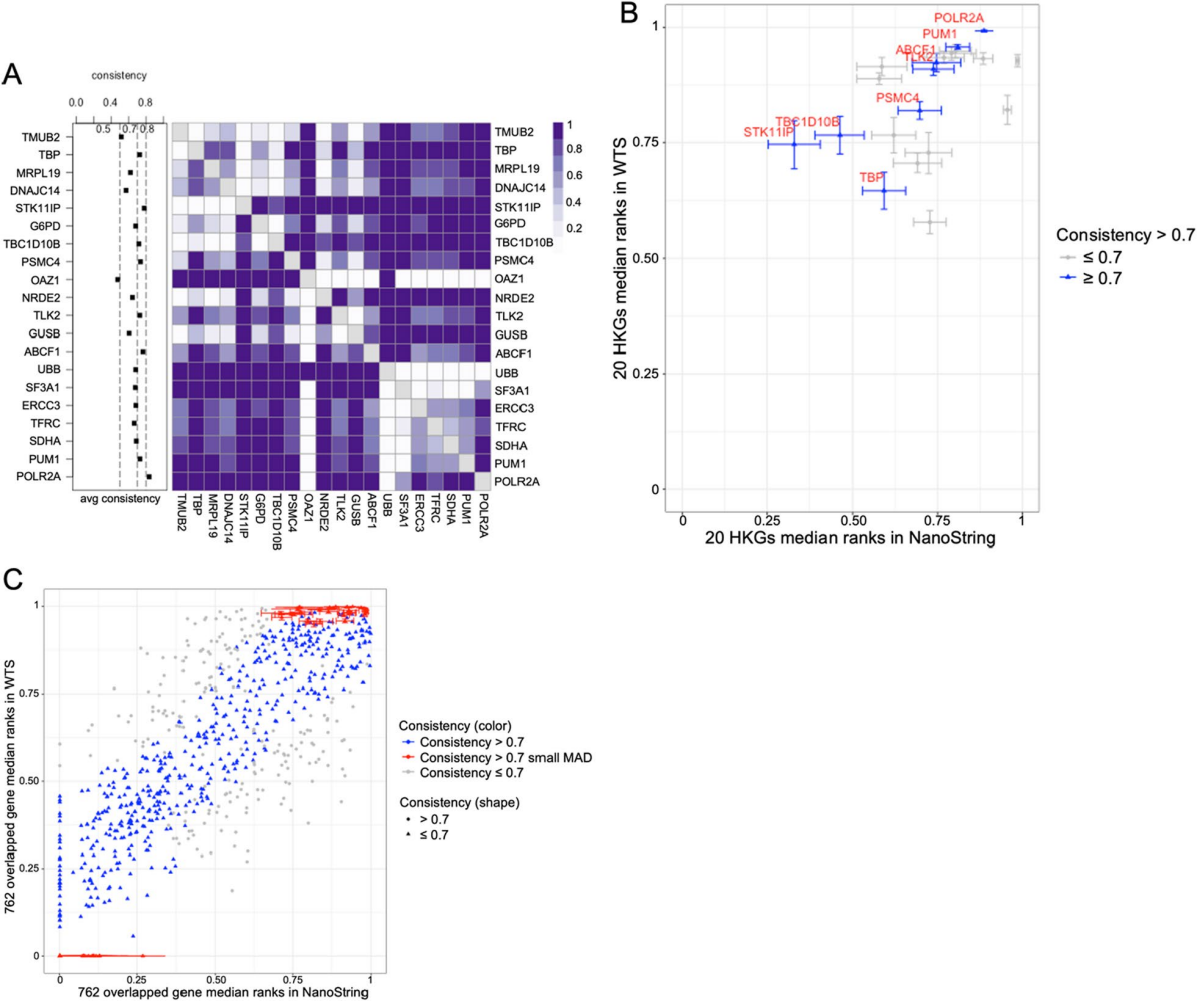
Rank consistency between NanoString and WTS platforms. **A** Cross-platform average consistency scores of 20 HKGs. The color bar indicates the pairwise consistency score. The darker colour in one cell represents a more consistent rank among the overlapping samples between this pair of genes. The left dot plot is each HKG’s average consistency. **B**, **C** The x- and y-axes are the median of the relative gene ranks in the NanoString and WTS platforms. The genes with average consistency scores > 0.7 in the NanoString platform are labelled. The horizontal and vertical error bars show the MAD of gene relative ranks in the NanoString and WTS platforms. **B** Rank locations and dispersions of 20 HKGs. **C** Rank locations of all 762 overlapping genes. The high consistency is average consistency score > 0.7; The high consistency, small MAD highlight the top 50 low MAD genes

We compared the expressional level similarity based on NanoString and WTS gene count data. For expression correlations, log_2_-transformed count data showed Spearman correlation (*r*) between 0.57 to 0.86 on 762 overlapping genes in homologous samples, and 33 out of 35 samples had *r* > 0.7 (Additional file 5: Table S13). For rank-based consistency, we first sought to examine the consistency of gene ranks, especially HKGs, across the NanoString and WTS platforms. The majority of the 20 HKGs used within the NanoString Pancancer 360 panel had robust expression patterns in both NanoString and WTS (Additional file 1: Fig. S6C, E). When referring to the rank, HKGs from NanoString had dispersed distribution, while the same HKGs from WTS were concentrated in the upper-half rank region with highly expressed portions and lower dispersions (Additional file 1: Fig. S6D, F). For the cross-platform rank consistencies of 20 HKGs, 8 HKGs displayed average consistency scores above 0.7. The highest consistency score was 0.84 in the *POLR2A* gene (Fig. 4A, Additional file 5: Table S14). Focusing only on the ranks of HKGs, they covered more rank regions in NanoString (25–100% rank region) compared to WTS (top 40% region) (Fig. 4B). Furthermore, to select potential cross-platform “stable genes”, the cross-platform rank consistency scores were measured on the overlapping 762 genes. Among them, 562 genes showed average consistency scores above 0.7 (Fig. 4C, Additional file 5: Table S15). More stably ranked genes were identified based on their Median Absolute Deviation (MAD) of the ranks among the samples. The top 50 smallest MAD genes gathered in the top and bottom quarter rank regions (Fig. 4C).

For the “Skewed ranks” method to measure *singscores* on NanoString platform, the cross-platform rank skewness was concerned. The median ranks of 770 genes in the NanoString platform and 22,297 genes in the WTS platform followed a relatively uniform distribution, lowly expressed gene (Additional file 1: Fig. S7A, B). The skewness in overlapping 762 genes can be observed that their ranks were concentrated at the median and high-rank regions in the WTS platform (Additional file 1: Fig. S7C). When fitting median ranks of the overlapping 762 genes in the WTS platform against a uniform distribution, a linear relation was revealed except for the bottom 20% of the lowly expressed genes (Additional file 1: Fig. S7E). This trend was consistent even if fitting regression in separate response groups (Additional file 1: Fig. S7F, G).

### Cross-platform *singscores* consistencies

We performed four pairs of comparisons to evaluate the similarity of cross-platform *singscores*.

**Fig. 5:**
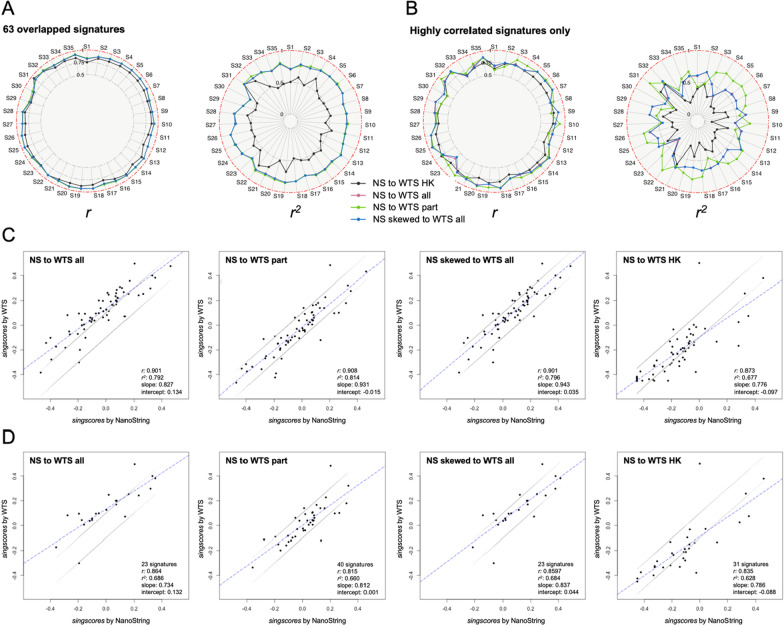
Cross-platform *singscore* consistency. **A**, **B** Radar plots of Spearman correlation (*r*), *r*^*2*^, of 35 overlapping samples (S1-35). The pairs of comparisons are labelled as different. The red dot-dashed circle line in each subplot represents the theoretical value when cross-platform *singscores* are identical in overlapping samples. **A** The left two plots display values based on 63 signatures, and (**B**) the right two display values from highly correlated signatures (*r* ≥ 0.8). 23 signatures were included in the “NS to WTS all” and “NS skewed to WTS all” comparison pairs, 40 signatures were chosen for the “NS to WTS part” comparison, and 31 signatures were chosen for the “NS to WTS HK” comparison. **C**, **D** The dot plots display consistency signatures’ *singscores* using sample S21 as an example. The four subplots are divided by four pairs of comparisons. The blue dotted line represents linear regression line. **C** all 63 signatures; **D** highly correlated signatures (*r* ≥ 0.8) only

All samples displayed highly correlated (*r* > 0.7) *singscores* between the NanoString and WTS platforms in all pairs of comparisons when using all signature scores. The three comparisons, “NS to WTS all genes”, limited to just the overlapping genes “NS to WTS part”, and “NS skewed to WTS all genes”, have high and similar correlations *r* (IQR [0.88, 0.92]) and *r*^*2*^ (IQR [0.77, 0.81]) (Fig. 5A). The “NS to WTS all” comparison had overall higher intercepts and lower slopes compared to “NS to WTS part” and “NS skewed to WTS all”. The “NS to WTS HK” comparison displayed lower *r*, *r*^*2*^, slope, and intercept values in all overlapping samples (Fig. 5C, Additional file 1: Fig. S8, Additional file 6: Table S17). When only focusing on the highly correlated signatures (per signature *singscores* correlation: *r* ≥ 0.8) (Additional file 6: Table S16), the “NS to WTS all” and “NS skewed to WTS all” comparison methods showed similar *r* (IQR [0.76, 0.87]) and *r*^*2*^ (IQR [0.53, 0.64]), while the “NS to WTS part” method had generally higher scores in these two values (*r*: IQR [0.75, 0.89]; *r*^*2*^: IQR [0.53, 0.74]) (Fig. 5B). The “NS to WTS all” comparison consistently displayed a higher intercept and lower slope compared to the others. The “NS to WTS HK” comparison method generated worse *r*, *r*^*2*^, slopes and intercepts when only focusing on the highly correlated signatures (Fig. 5D, Additional file 1: Fig. S9, Additional file 6: Table S18).

At the signature level, the “NS to WTS all” and “NS skewed to WTS all” comparison methods provided the same list of highly correlated signatures (23 signatures with *r* ≥ 0.8), and the “NS to WTS part” method displayed more highly correlated signatures (40 signatures with *r* ≥ 0.8) (Additional file 6: Table S16). Based on the number of signatures, those with more genes were more likely to generate consistent *singscores* in the cross-platform study (Additional file 1: Fig. S10B, C).

### Cross-platform predictions using *singscores*

**Fig. 6:**
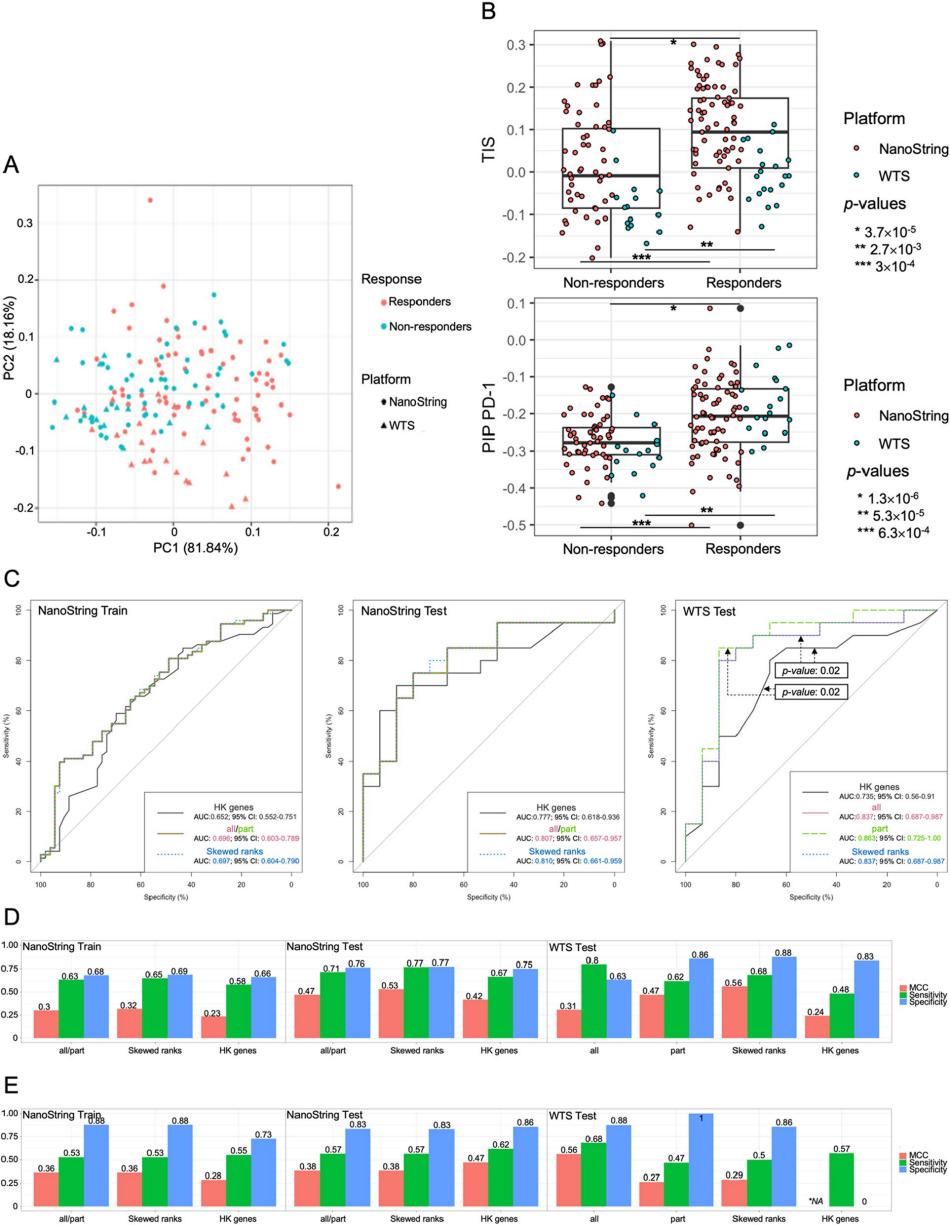
Cross-platform predictions. **A** PCA plot and (**B**) boxplot on *singscores* for two selected signatures: TIS and PIP PD-1, for all 161 samples (126 NanoString training samples, 35 WTS testing samples). The *singscores* were calculated by the “No stable gene” method in the NanoString platform and the “part” method in the WTS platform. The dots are colored by the platform (NanoString or WTS). The *p*-values were measured by *Mann Whitney* Wilcoxon test. **C** ROC curves of predicting NanoString and WTS samples using logistic regression models. Due to using the same model, the “all” and “part” have identical ROC curves when predicting samples from NanoString platform. The colors differentiate the pairs of comparisons. **D**, **E** MCC, sensitivity and specificity values of binary clustering based on the predictive probability from different logistic regression models. **D** using default threshold (probability of response ≥ 0.5 as responder); **E** using optimal threshold (maximizing Youden index in ROC curve from training samples) in each model. NA in sensitivity was due to no sample being classified as responder

To evaluate the utility of the good-performing highly correlated *singscores,* we assessed their ability to classify the responders from the non-responders. The *singscores* from different calculating methods in 126 NanoString training samples were applied in the LASSO regression to select signatures, which were further used in building the logistic regression model and then tested in predicting the probability of response of the 35 overlapping samples in the NanoString and WTS platforms. During the feature selection on the *singscores* from “No stable gene” and “Skewed rank” methods, the TIS and PIP PD-1 were the top two frequently selected signatures in the most repeats, while the IFNg-6 was the commonly selected signature in the “HK genes” method (Fig. 1B, Additional file 1: Fig. S11). When focusing on TIS and PIP PD-1 signatures, samples tended to form separate clusters based on different response statuses (Fig. 6A) and displays significantly higher values in responders in both platforms (Fig. 6B).

When predicting training and testing datasets from NanoString platform, the predictive ROC curves provided similar AUC values among logistic regression models based on three different training *singscore* tables (Fig. 6B right, middle). The model generated by the “HK genes” method displayed a significantly (*p*-value = 0.02) low AUC (73.5%) then the values from other methods (“part”: 86.3%; “Skewed ranks” and “all”: 83.7%) when predicting overlapping WTS samples (Fig. 6C left).

When predicting response status using the default probability threshold, predictive performances (MCC, sensitivity and specificity) of testing datasets from NanoString samples were similar in three different logistic regression models. In the performance of predicting the overlapping WTS sample, the models built on *singscores* from “No stable gene” method to predict WTS samples by “part” method, and “Skewed ranks” method to predict WTS samples by “all” method provided better predictions (MCC = 0.47 and 0.56). The model built on *singscores* from “No stable gene” method to predict WTS samples by “all” method classified the majority of WTS samples as responders, and the *singscores* from the “HK genes” methods generated a model that misclassified many WTS samples into non-responders (Fig. 6D, Additional file 6: Table S19).

Differentiated from the default threshold, the optimal thresholds are different in three logistic regression models. All models suggested a stricter threshold to predict a sample as a responder (Additional file 6: Table S19). By applying optimal thresholds, all models displayed more likely to misclassify true responders as non-responders (higher specificity) than using the default threshold in the majority of predictions. The model built on *singscores* from “No stable gene” method has better prediction of WTS samples by “all” method when using the optimal threshold (Fig. 6E).

## Discussion

This study found that *singscore*-based signature scores were highly reproducible across replicates, and consistent even after normalisation. The use of *singscore* generated highly correlated and reproducible scores across 12 repeated samples generated from different batches of cartridges. Likewise, although the normalisation and background thresholding may change low-expression gene ranks, *singscore* still provided stable scores with or without the HKGs normalisation function, geNorm [26], during the nSolver sample normalisation steps. Moreover, *singscore*-derived signature scores could even be used in cross-platform analysis. High correlation values were observed in the majority of WTS samples. Overall, we validated the utility of the approach to reproducibility in identifying patients likely to respond to anti-PD-1-based immunotherapies based on NanoString gene expression data.

Our study explored rank-based signature scores in interpreting differences in response status. Within the 81 signatures, the significantly (FDR ≤ 0.05) different signatures included multiple immune-related and cell signalling pathways. Many PD-1, T-cell, and IFN-γ-related genes and signatures showed significantly high levels in responders, which correspond to preferable response in immunotherapies [3, 4, 10, 14, 27]. Reverse scoring trends were observed in the signatures, including angiogenesis, hypoxia and WNT signalling, which were reported to be inversely correlated to response [3] and to drug resistance [27]. Additionally, *singscore*-derived signatures were highly reproducible within the NanoString replicates, as well as between raw and normalised samples. Therefore, using the rank-based signature scores from NanoString is a reliable and representative approach to identify the differences in response status.

As a highly sensitive, reproducible, and robust technique for evaluating targeted gene expression profiles, our NanoString assay also reported high correlations to WTS data in matched samples in the log-transformed count data [5]. Differential expression analysis of the NanoString data identified similar lists of differential expression genes (DEGs) to the analysis performed from our previous WTS study [3]. Within 549 DEGs identified in the WTS study, 115 DEGs exist in NanoString probes. Half of them were also reported as DEGs in the NanoString assay (56 adj. *p*-values ≤ 0.05, 74 *p*-values ≤ 0.05). Many signature genes highlighted in the WTS study were also upregulated in responders with an adjusted *p*-value ≤ 0.05 (IFN-γ-related genes: *STAT1*, *IRF1*; T cell infiltration and cytotoxicity related genes: *PDCD1*, *CXCL13*, *CD2*, *CD247*, *CD274*, *CD5*, *CD6*; Cytokine signalling: *CCL4*, *CCR5*, *CXCL9*, *CXCL13*; Immunosuppressive checkpoints: *TNFRSF9*, *IDO1*, *LAG3*). Although genes in the NanoString nSolver in-built pathways in our study differed from the genes in KEGG pathways used in the WTS study, some significantly different pathways reported in nSolver analysis were also found in the WTS study, such as Cytotoxicity, JAK-STAT Signalling, and Cytokine and Chemokine Signalling [3].

In cross-platform comparisons, highly correlated *singscores* also support the robustness and reproducibility of *singscore*. Most of the NanoString nCounter^®^ PanCancer IO 360^™^ genes are intermediate and high expression genes compared to the WTS ranked profiles. It resulted in that *singscores* derived from the NanoString platform were generally lower scores under the same scoring method from WTS. While highly correlated, the skew in gene expression distribution will lead to different *singscores* between platforms and be problematic in some cross-platform analyses, such as model building and prediction. Our study suggests two possible solutions to alleviate this impact. One is to subset the WTS data to analyse only the overlapping genes (“part” method). The other way is to modify the ranks in the NanoString platform by fitting the raw ranks using linear regression coefficients (“Skewed ranks” method). Many samples’ ranks follow a uniform distribution. However, some samples contained more low signal probes (raw count ≤ default background noise 20). The transformation did not change their ranks (rank always 0 before and after skewing ranks). Although the “Skewed ranks” method provided highly correlated and similar cross-platform *singscores* and good prediction, it is not a generalisable method. When crossing multiple expression datasets or another dataset with a different number of genes, the coefficients for skewing need to be adjusted per assay. Therefore, focusing only on the overlapping genes may be a simpler and more feasible option. Additionally, although using a different probability threshold to predict binary responding outcomes (optimal threshold) may alleviate this problem, it also leads to a higher risk to misclassify the true responders as non-responders.

Another intuitive method to overcome this cross-platform *singscore* variation was to introduce “stable genes” to calibrate across samples. Through analysing the perseveration of gene ranks across multiple platforms, Bhuva *et.al* introduced a list of stable genes, which is an in-built function in *singscore* [25]. However, only 2 out of the top 20 in-built stable genes were found in the NanoString 360 IO probe set. If only using these two genes to calibrate, it may underestimate signature scores and veil the differences between the response status. Alternatively, we tried to apply the 20 HKGs from the NanoString probe set as stable genes. Unfortunately, the subsequent results were less informative in the differences between response and non-response, decreasing overall signature scores, and poor cross-platform correlations. HKGs’ rank distributions and inconsistency in cross-platform ranks may explain this problem. Although these HKGs displayed robust expressions and ranks in the WTS platform, their ranks were concentrated in the upper half region of expression. Accurate stable genes should have a high-rank consistency which means the orders among the stable genes should be persevered across platforms [25]. However, nearly all HKGs showed lower average consistency scores compared to the recommended stable genes in *singscore*. Additionally, this study attempted to identify platform-specific “stable genes” using a similar idea on all 762 overlapping genes. However, no suitable stable gene for cross-platform calibration could be identified between the NanoString Pancancer 360 IO panel and WTS. Therefore, when choosing the cross-sample stable genes for *singscore*, the stability in gene expression is not a good selective criterion. Checking the distribution and preservation of consistency in cross-platform ranks are important before introducing any stable gene for calibration.

The LASSO regression was used to select more informative signatures and to test the clinical utility of the *singscore* based signatures in predicting immunotherapy response. Although such prediction was still not highly accurate, it can be further improved by combining other information, including tumour mutation burden [4, 28] and clinical features [29]. The PIP PD-1 signature and TIS were two the predominant features selected during the repeated training. They were also in the top-3 signatures where responders have significantly higher *singscores*. The consistent tendency can still be observed when crossing platforms. Site of biopsy used to generate the subsequent *singscores* should be considered within cohorts prior to model building as some signatures can differ significantly based on the tissue or origin. While the lymph node specimens displayed significantly different scores in multiple signatures compared to other biopsy sites, there were no statistically significant differences in the PIP PD-1 and TIS signatures between sites and or any association with responses. The predictive MCC indicated the good power of these two signatures in classifying response status in the testing datasets. Higher PIP PD-1 and TIS scores were observed in some non-responders, resulting in the internal prediction on training samples misclassifying them as responders. These patients may have immune-excluded tumours, with immune cells enriched in the stroma surrounding the tumour region but lacking infiltration in the intratumoural region [30, 31]. The PIP PD-1 signature, an in-house derived signature, contained three genes, *PDCD1*, *PDCD1LG2*, *CD274*. Differential expression results in nSolver also labelled these genes as significantly highly expressed in responders. Among them, the PD-L1 (*CD274*) gene expression level is a validated biomarker for anti-PD-1 monotherapy and anti-PD-1 + anti-CTLA-4 in advanced melanoma [32, 33]. TIS is an 18-genes signature containing genes relating to antigen presentation, IFN signalling, and T-cell and NK cell activities [14, 34]. The higher TIS scores were observed in responding patients in our study, which is consistent with previous studies showing an association between high TIS scores and better overall survival and response to anti-PD-1 monotherapy [34, 35]. The consistency and capability of TIS to be a potential biomarker for tumour inflammation and response to anti-PD-1 therapy on the NanoString platform have also been validated [36, 37].

## Conclusion

Consistent with our previous publication [3], we demonstrate that NanoString nCounter^®^ PanCancer IO 360^™^ can generate a similar immune profile to that generated by the WTS platform in advanced melanoma patients, and illustrate that the rank-based scoring tool, *singscore*, is also a reliable and practical approach to analyse the variations of immune signatures between response status and conduct cross-platform analysis.

## Supplementary Information

**Additional file 1: Figure S1.** Expressional distributions of samples from NanoString and WTS assays; **Figure S2.** Heatmap of NanoString raw and normalized counts data; **Figure S3.** PCA plot of NanoString samples; **Figure S4.** Heatmap of *singscores* of significant signatures on NanoString samples by the “No stable gene”, “Skewed ranks”, and “HK genes” methods; **Figure S5.** Linear regression of NanoString repeats; **Figure S6.** Boxplot of HKGs expressions and ranks in NanoString and WTS samples; **Figure S7.** Plots of rank distributions of all and overlapping genes in NanoString and WTS platforms; **Figure S8, S9.** Linear regressions of overlapping samples’ *singscores* based on all signatures and highly correlated signatures; **Figure S10.** Signature level *singscores* consistency; **Figure S11.** Frequency of selected signatures by LASSO regression in 1000-time repeats.

**Additional file 2: Table S1.** All NanoString samples metadata; **Table S2.** NanoString sample names map to the overlapping WTS sample names; **Table S3.** Repeating samples (derived from same patients) in NanoString assay; **Table S4.** Repeating Details of modification in WTS genes to match NanoString probes; **Table S5.** Details of Immune signatures used in this study.

**Additional file 3: Table S6**–**S8.** *Mann Whitney* Wilcoxon test significance of signatures between response status on NanoString samples using signature scores from different singscore calculating methods. **Table S9.** *Mann Whitney* Wilcoxon test significance of signatures between site of biopsy on the lymph node or not.

**Additional file 4: **Results of NanoString samples using advanced analysis in nSolver4.0. **Table S10.** Differential expression analysis on response; **Table S11.** Pathway scores. **Table S12.** *Mann *Whitney Wilcoxon test significance of signatures between response status on NanoString samples using nSolver pathway scores.

**Additional file 5: Table S13.** Cross-platform Spearman correlations of 35 overlapping samples counts data; **Table S14**, **S15.** Gene rank consistency tables of 20 HKGs and all 762 overlapping genes.

**Additional file 6: Table S16.** Cross-platform per signature Spearman correlations of *singscores* using different calculating methods; **Table S17**, **S18.** Cross-platform linear-regression coefficients and Spearman correlations of all and highly correlated signatures’ *singscores* using different calculating methods; **Table S19.** Confusion matrixes of cross-platform response predictions by logistic regression models using frequently selected signatures.

## Data Availability

The NanoString RCC data that support the findings of this study are available from European Genome-phenome Archive (EGA study Accession Number: EGAS00001006977) but restrictions apply to the availability of these data, which were used under license for the current study, and so are not publicly available. Data are however available from the authors upon reasonable request and with permission of Melanoma Institute Australia (MIA).

## Abbreviations

AUC: Area under the curve
DEG: Differential expression genes
FDR: False discovery ratio
FFPE: Formalin-fixed paraffin-embedded
HKG: House keeping gene
IQR: Inter quartile range
LASSO: Least absolute shrinkage and selection operator
MCC: Matthews correlation coefficient
MAD: Median absolute deviation
NS: NanoString
PIP: Personalised immunotherapy platform
QC: Quality control
TIS: Tumour inflammation signature
WTS: Whole transcriptome sequencing
