# Supplementary material for: Cross-platform comparison of immune signatures in immunotherapy-treated patients with advanced melanoma using a rank-based scoring approach
Source: J Transl Med. 2023 Apr 13;21:257. doi: 10.1186/s12967-023-04092-9 (PMC10103529; doi:10.1186/s12967-023-04092-9)
Supplement: Supplementary file 1 — Additional file 1: Figure S1. Expressional distributions of samples from NanoString and WTS assays; Figure S2. Heatmap of NanoString raw and normalized counts data; Figure S3. PCA plot of NanoString samples; Figure S4. Heatmap of singscores of significant signatures on NanoString samples by the “No stable gene”, “Skewed ranks”, and “HK genes” methods; Figure S5. Linear regression of NanoString repeats; Figure S6. Boxplot of HKGs expressions and ranks in NanoString and WTS samples; Figure S7. Plots of rank distributions of all and overlapping genes in NanoString and WTS platforms; Figure S8, S9. Linear regressions of overlapping samples’ singscores based on all signatures and highly correlated signatures; Figure S10. Signature level singscores consistency; Figure S11. Frequency of selected signatures by LASSO regression in 1000-time repeats. [file 12967_2023_4092_MOESM1_ESM.pdf]

Additional file

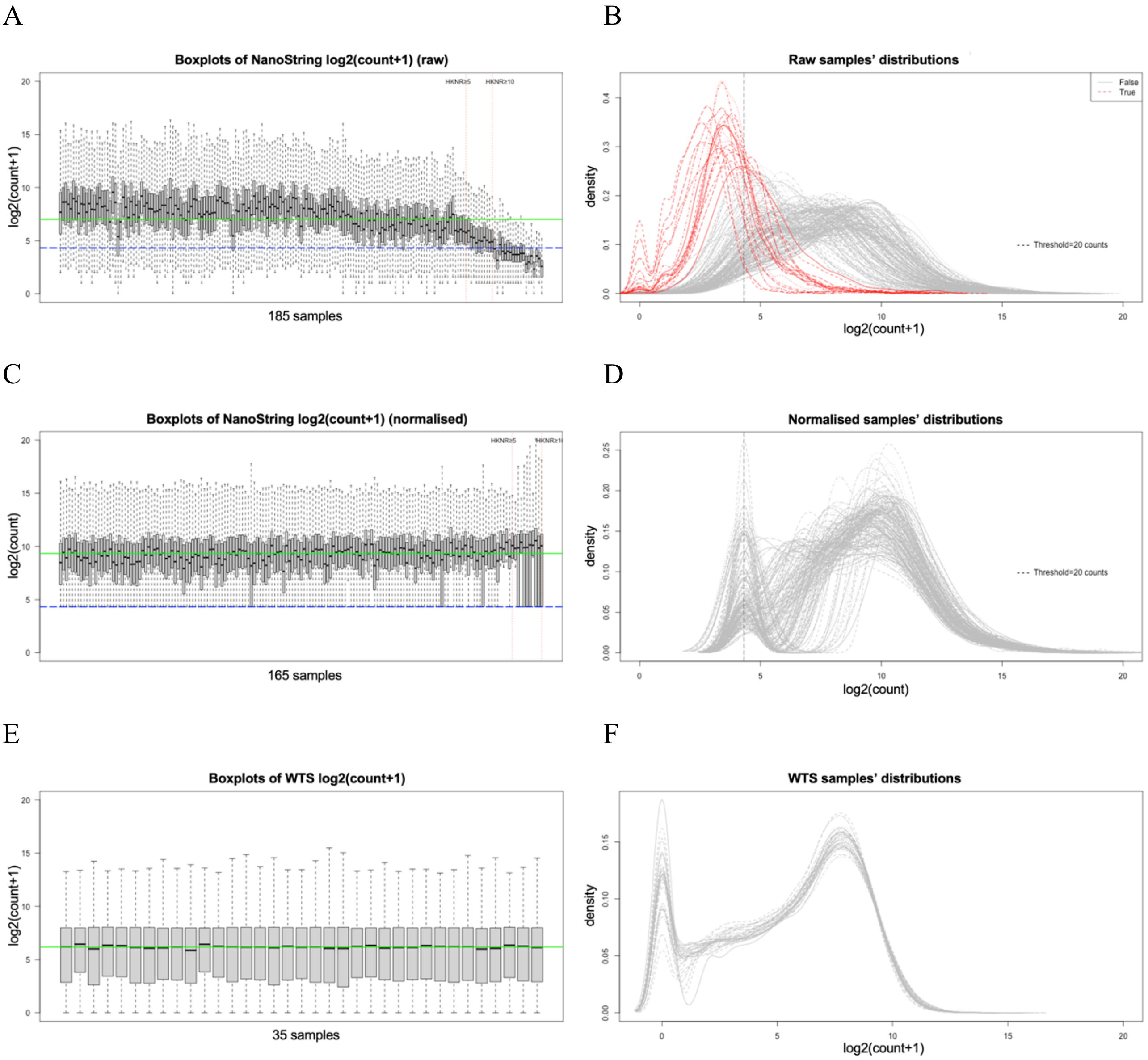

Figure S1. Distribution plots of NanoString and WTS samples. (A-D) Sample distribution plots on NanoString expression data. **(E-F)** Sample distribution plots on WTS expression data. **(A,C,E)** Each boxplot represents one sample. The y-axis is $\log_2$-transformed count values. The horizontal green line represents the median expression value across all samples. The horizontal blue dashed line is $\log_2(20)$, which is defined as background noise in NanoString data. The two vertical dotted lines stand for housekeeping gene normalisation ratio ≥ 5 (left) and ≥ 10 (right). Samples in NanoString boxplots are ordered by the housekeeping gene normalisation ratio in an ascending order from the left to the right. **(A)** Boxplot on raw NanoString expression data. **(C)** Boxplot on normalised NanoString expression data. **(E)** Boxplot on WTS expression data. **(B,D,F)** Each curve represents one sample. The x-axis is $\log_2$ transformed count values. The vertical black dashed line is $\log_2(20)$. **(B)** Line plot on raw NanoString expression data. The red lines represent samples with housekeeping gene normalisation ratio ≥ 10. **(D)** Line plot on normalised NanoString expression data. Sample with housekeeping gene normalisation ratio ≥ 10 already filtered out. **(F)** Line plot on WTS expression data.

A

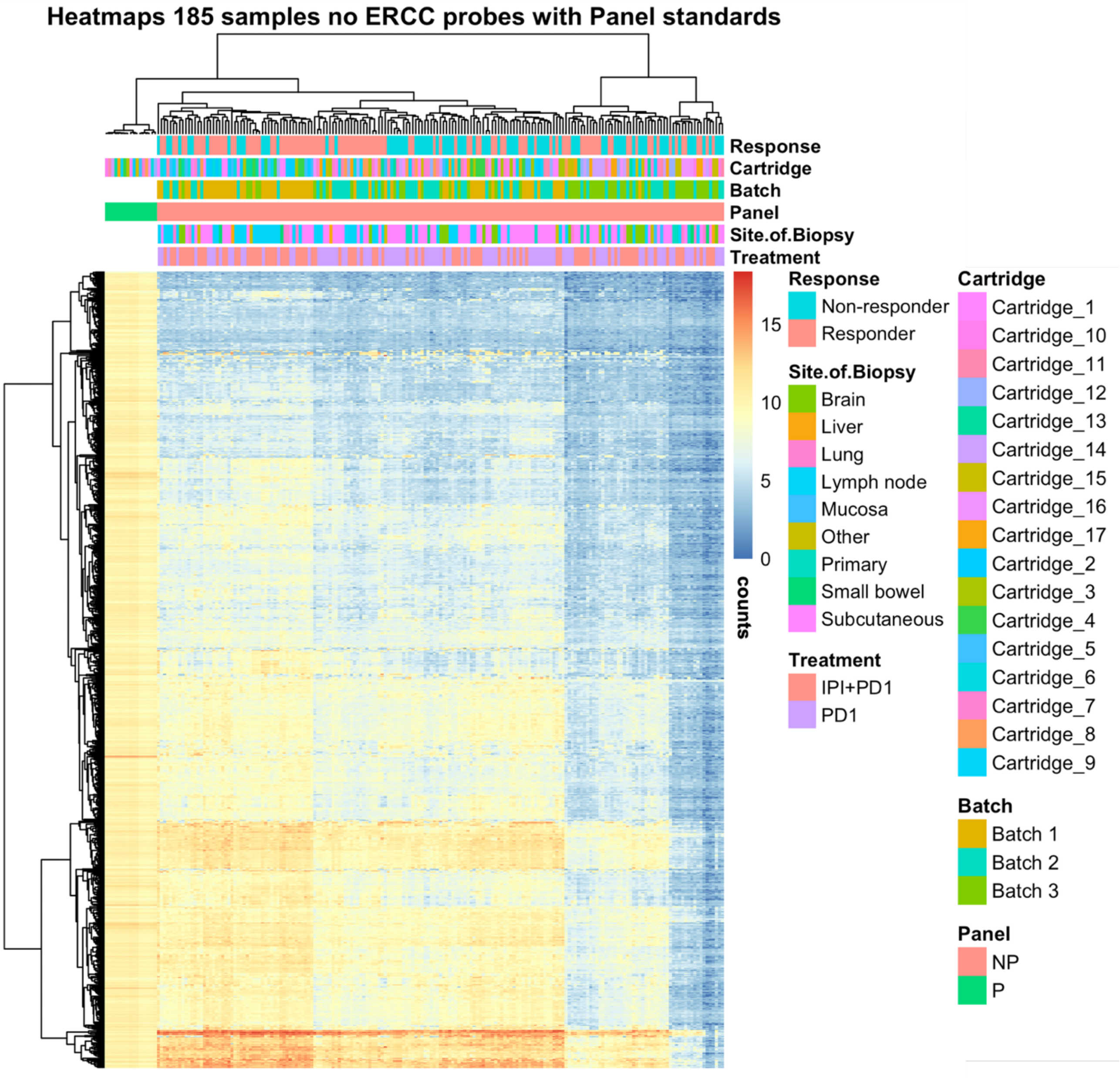

Heatmaps 185 samples no ERCC probes with Panel standards
Response
Cartridge
Batch
Panel
Site.of.Biopsy
Treatment
15
10
5
0
counts
Response
Non-responder
Responder
Site.of.Biopsy
Brain
Liver
Lung
Lymph node
Mucosa
Other
Primary
Small bowel
Subcutaneous
Treatment
IPI+PD1
PD1
Cartridge
Cartridge_1
Cartridge_10
Cartridge_11
Cartridge_12
Cartridge_13
Cartridge_14
Cartridge_15
Cartridge_16
Cartridge_17
Cartridge_2
Cartridge_3
Cartridge_4
Cartridge_5
Cartridge_6
Cartridge_7
Cartridge_8
Cartridge_9
Batch
Batch 1
Batch 2
Batch 3
Panel
NP
P

B

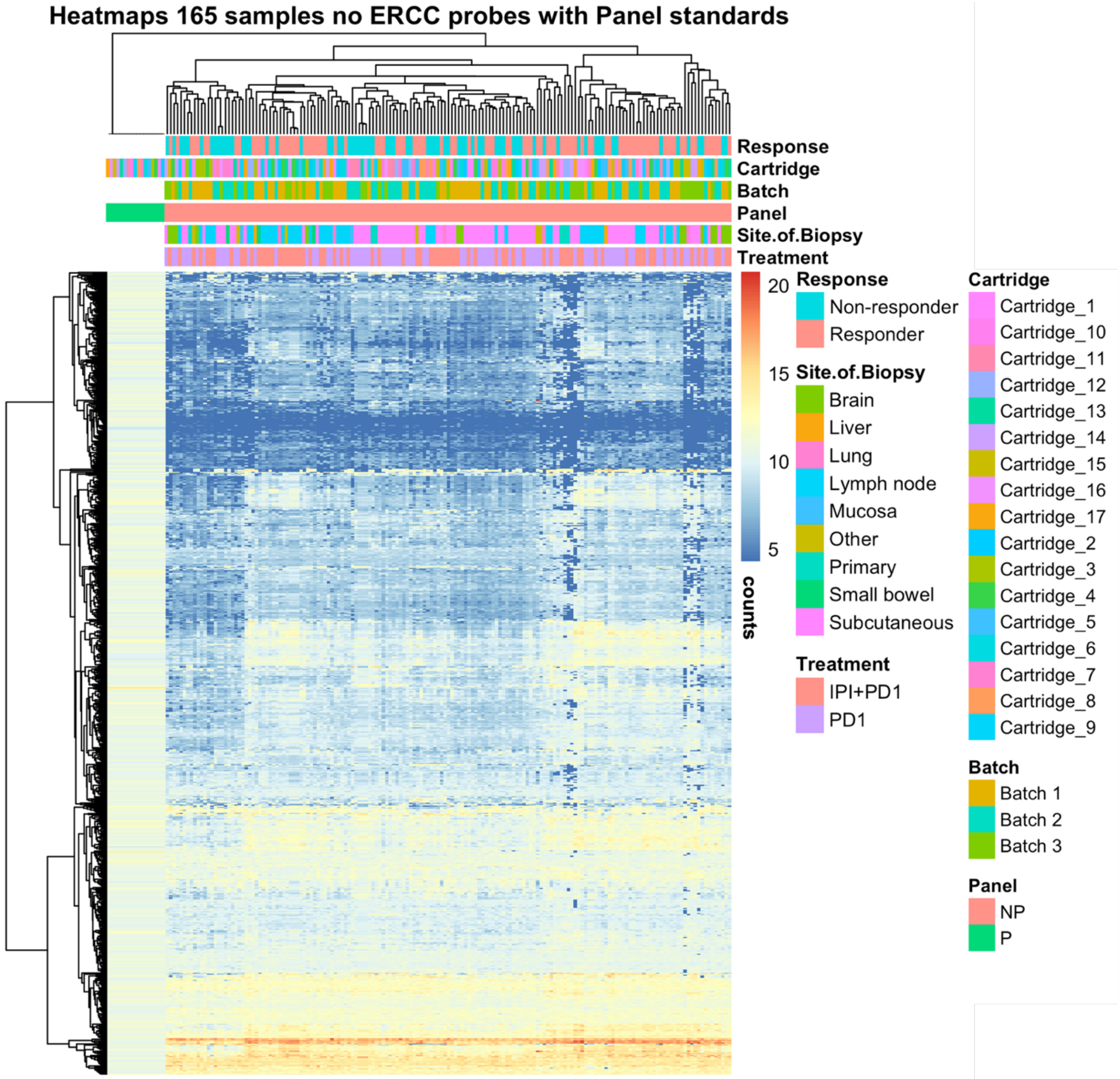

Figure S2. Heatmap of NanoString counts. The heatmap shows the samples' raw counts. Hierarchical clustering group on samples (columns) and genes (rows). Clinical features are labelled for each sample. "NP" and "P" in Panel category distinguish sample count and Panel standard count. 14 Engineered RNA sequences (ERCC) probes (6 positive controls and 8 negative controls) were removed, and only mRNA probes were kept in this figure. The color bar is based on the log_2-transformed count values. **(A)** 185 samples' raw counts. **(B)** 165 samples' normalised counts.

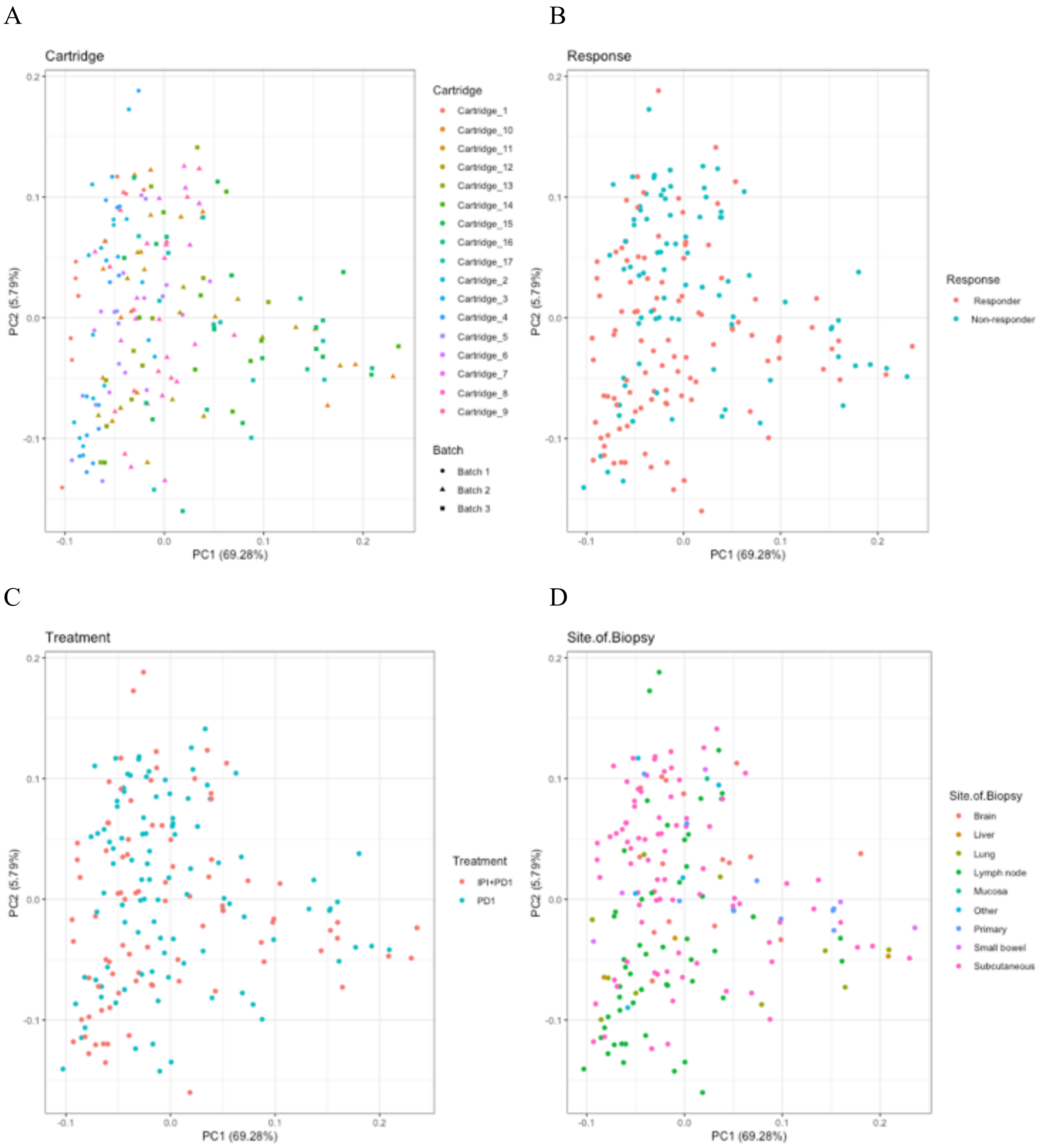
A
Cartridge
Cartridge
Cartridge_1
Cartridge_10
Cartridge_11
Cartridge_12
Cartridge_13
Cartridge_14
Cartridge_15
Cartridge_16
Cartridge_17
Cartridge_2
Cartridge_3
Cartridge_4
Cartridge_5
Cartridge_6
Cartridge_7
Cartridge_8
Cartridge_9
Batch
Batch 1
Batch 2
Batch 3
PC1 (69.28%)
PC2 (5.79%)
B
Response
Response
Responder
Non-responder
PC1 (69.28%)
PC2 (5.79%)
C
Treatment
Treatment
IPI+PD1
PD1
PC1 (69.28%)
PC2 (5.79%)
D
Site.of.Biopsy
Site.of.Biopsy
Brain
Liver
Lung
Lymph node
Mucosa
Other
Primary
Small bowel
Subcutaneous
PC1 (69.28%)
PC2 (5.79%)

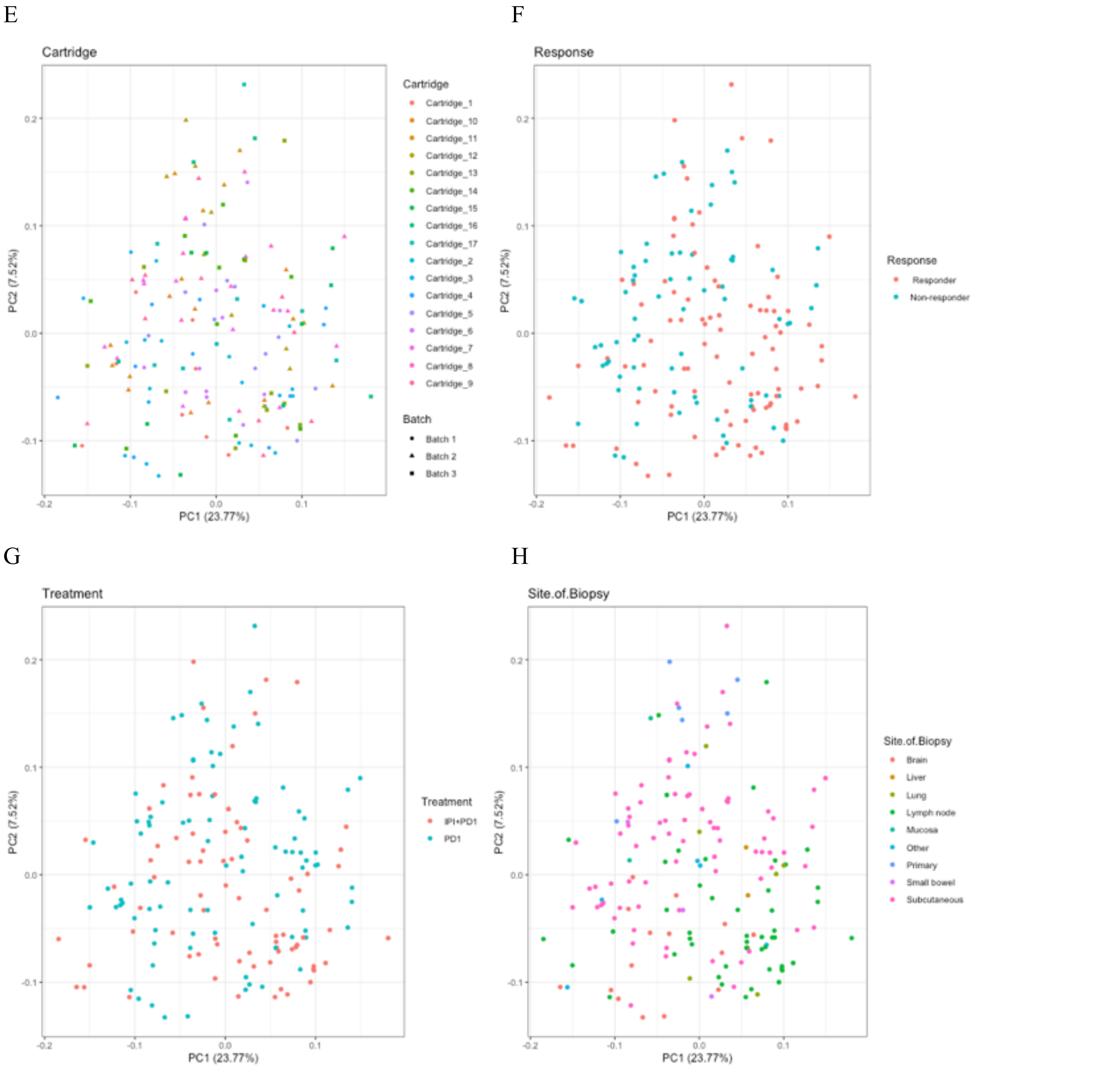

Figure S3. PCA plots on 165 NanoString samples. (A-D) on the raw counts data. **(E-H)** on the normalised counts data. **(A, E)** samples are colored by cartridges, and labelled by batches. **(B, F)** samples are colored by response status. **(C, G)** samples are colored by immunotherapies. **(D, H)** samples are colored by the sites of biopsy.

A

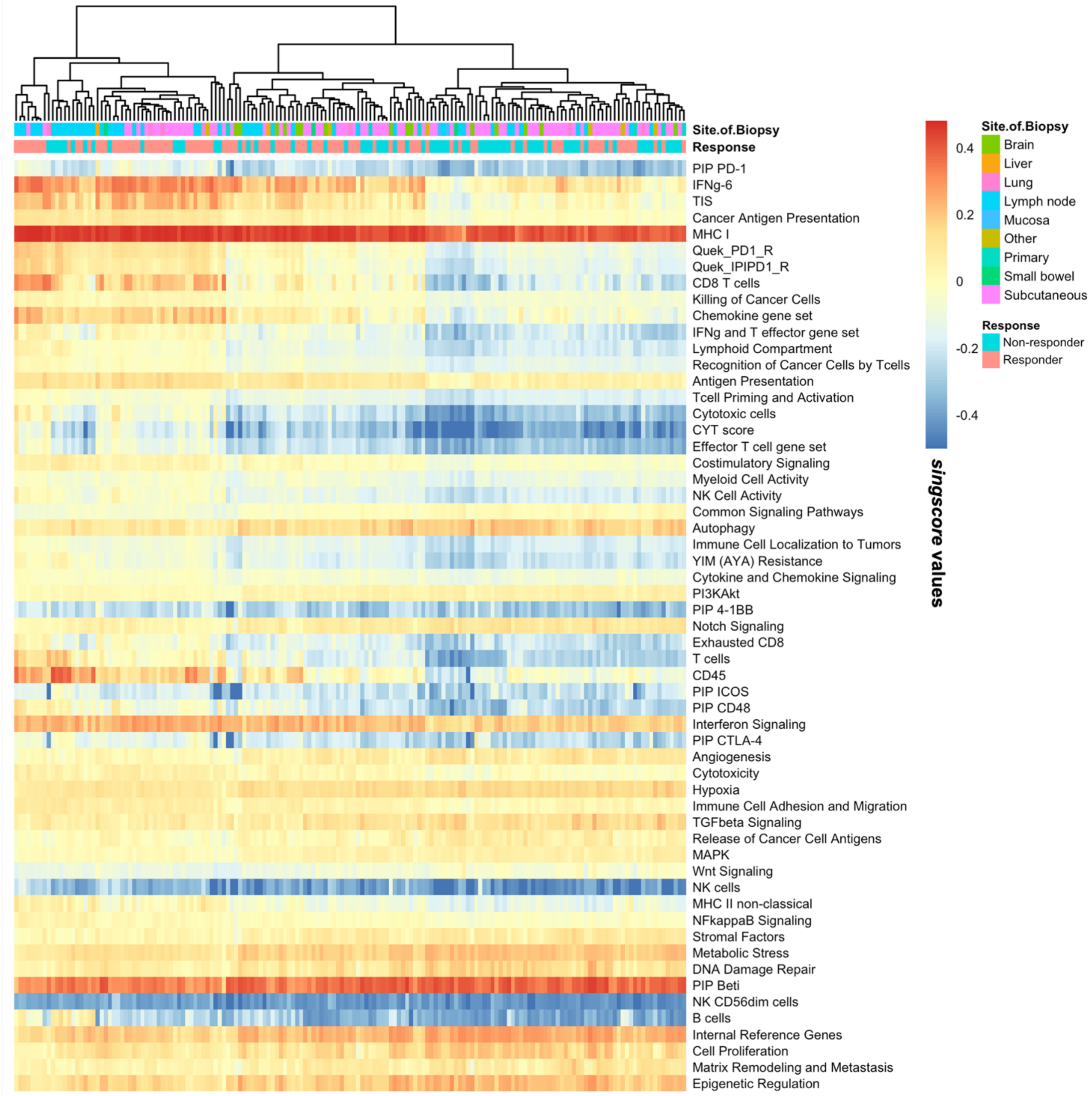

Site.of.Biopsy
Response
PIP PD-1
IFNg-6
TIS
Cancer Antigen Presentation
MHC I
Quek_PD1_R
Quek_IPIPD1_R
CD8 T cells
Killing of Cancer Cells
Chemokine gene set
IFNg and T effector gene set
Lymphoid Compartment
Recognition of Cancer Cells by Tcells
Antigen Presentation
Tcell Priming and Activation
Cytotoxic cells
CYT score
Effector T cell gene set
Costimulatory Signaling
Myeloid Cell Activity
NK Cell Activity
Common Signaling Pathways
Autophagy
Immune Cell Localization to Tumors
YIM (AYA) Resistance
Cytokine and Chemokine Signaling
PI3KAkt
PIP 4-1BB
Notch Signaling
Exhausted CD8
T cells
CD45
PIP ICOS
PIP CD48
Interferon Signaling
PIP CTLA-4
Angiogenesis
Cytotoxicity
Hypoxia
Immune Cell Adhesion and Migration
TGFbeta Signaling
Release of Cancer Cell Antigens
MAPK
Wnt Signaling
NK cells
MHC II non-classical
NFkappaB Signaling
Stromal Factors
Metabolic Stress
DNA Damage Repair
PIP Beti
NK CD56dim cells
B cells
Internal Reference Genes
Cell Proliferation
Matrix Remodeling and Metastasis
Epigenetic Regulation
0.4
0.2
0
-0.2
-0.4
singscore values
Site.of.Biopsy
Brain
Liver
Lung
Lymph node
Mucosa
Other
Primary
Small bowel
Subcutaneous
Response
Non-responder
Responder

B

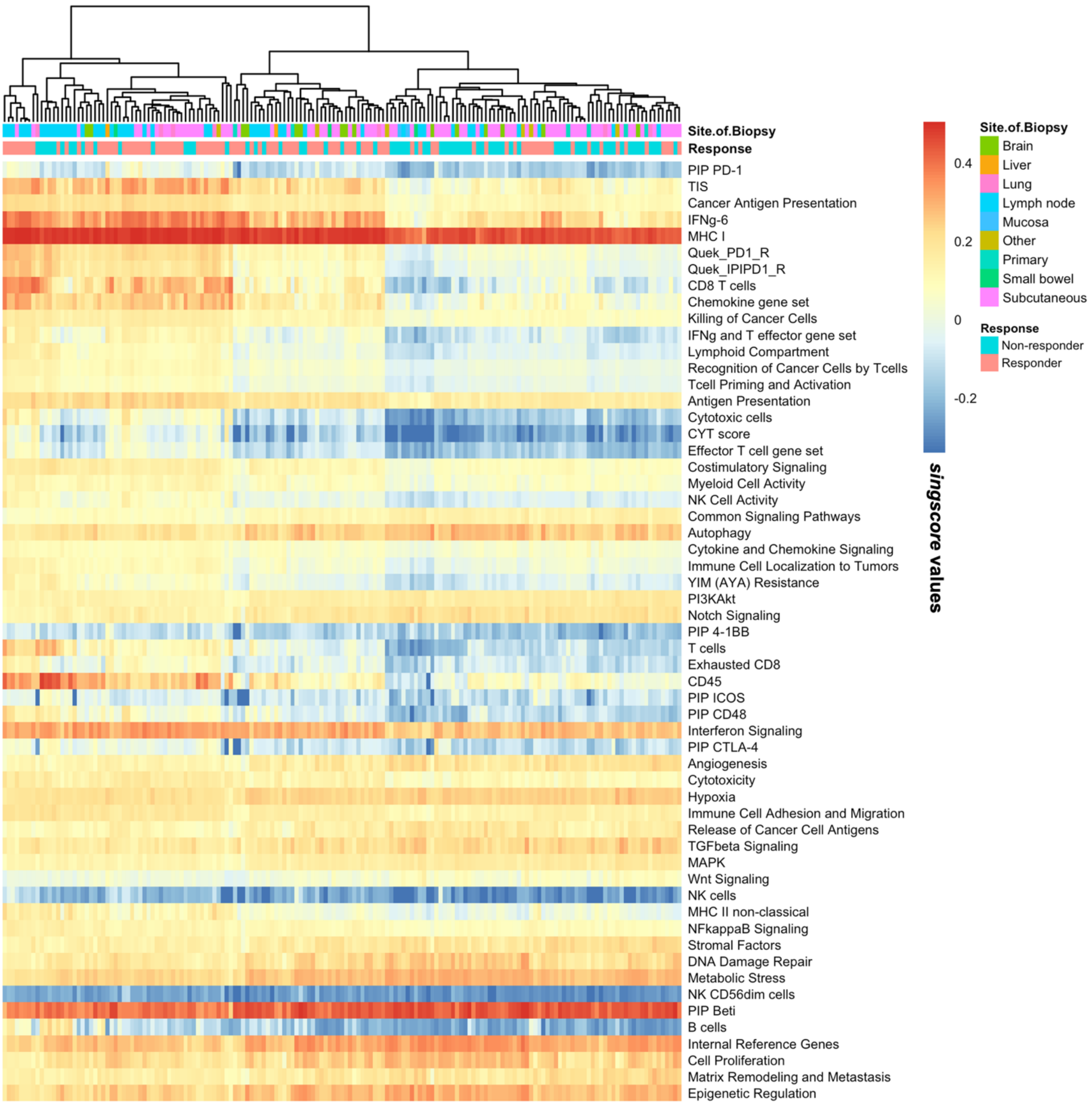
Site.of.Biopsy
Response
PIP PD-1
TIS
Cancer Antigen Presentation
IFNg-6
MHC I
Quek_PD1_R
Quek_IPIPD1_R
CD8 T cells
Chemokine gene set
Killing of Cancer Cells
IFNg and T effector gene set
Lymphoid Compartment
Recognition of Cancer Cells by Tcells
Tcell Priming and Activation
Antigen Presentation
Cytotoxic cells
CYT score
Effector T cell gene set
Costimulatory Signaling
Myeloid Cell Activity
NK Cell Activity
Common Signaling Pathways
Autophagy
Cytokine and Chemokine Signaling
Immune Cell Localization to Tumors
YIM (AYA) Resistance
PI3KAkt
Notch Signaling
PIP 4-1BB
T cells
Exhausted CD8
CD45
PIP ICOS
PIP CD48
Interferon Signaling
PIP CTLA-4
Angiogenesis
Cytotoxicity
Hypoxia
Immune Cell Adhesion and Migration
Release of Cancer Cell Antigens
TGFbeta Signaling
MAPK
Wnt Signaling
NK cells
MHC II non-classical
NFkappaB Signaling
Stromal Factors
DNA Damage Repair
Metabolic Stress
NK CD56dim cells
PIP Beti
B cells
Internal Reference Genes
Cell Proliferation
Matrix Remodeling and Metastasis
Epigenetic Regulation
0.4
0.2
0
-0.2
singscore values
Site.of.Biopsy
Brain
Liver
Lung
Lymph node
Mucosa
Other
Primary
Small bowel
Subcutaneous
Response
Non-responder
Responder

C

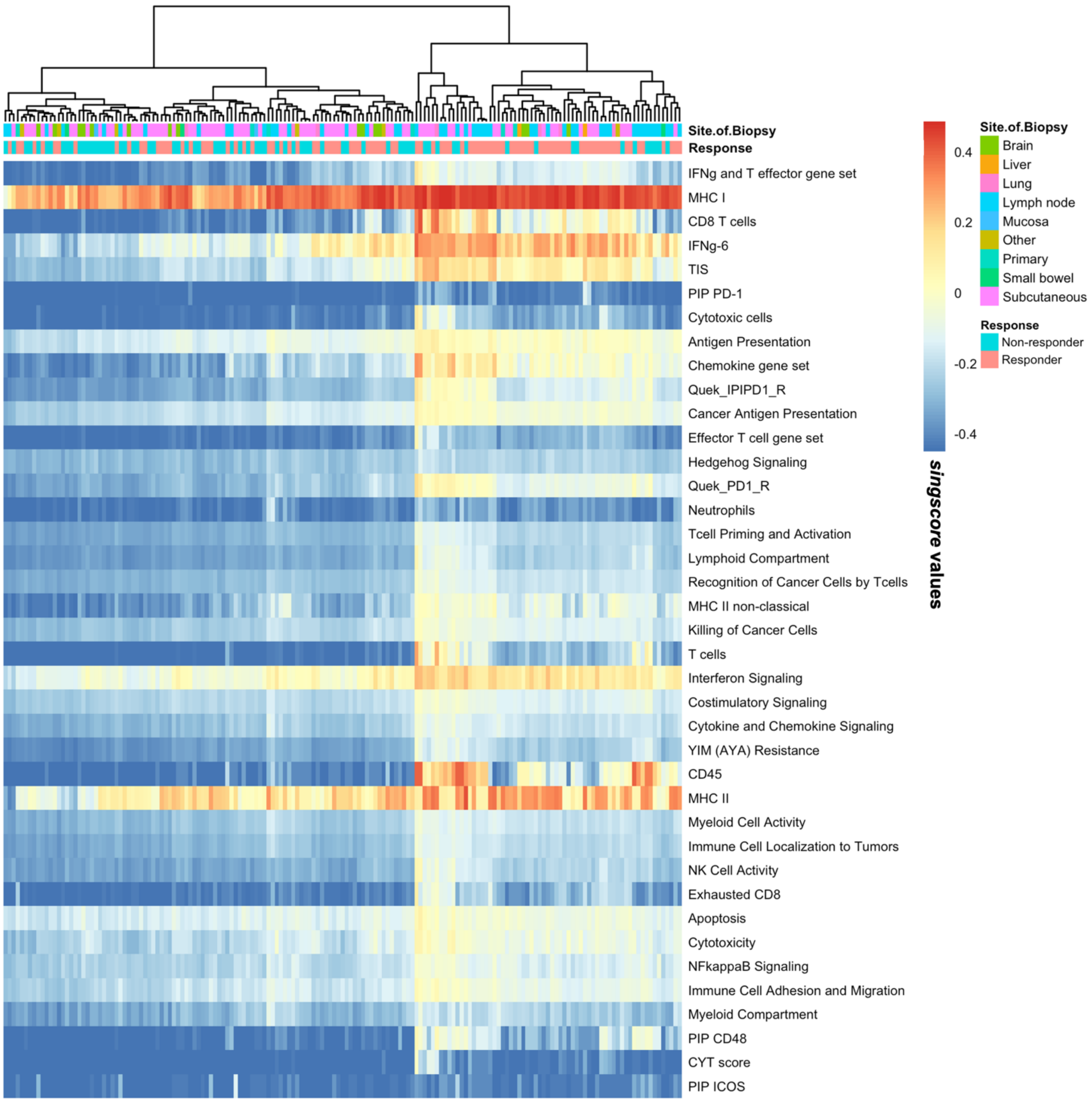

Figure S4. Heatmap of significant signatures. The signatures listed in each plot passed the FDR (*BH* adjustment on *Mann Whitney* Wilcoxon test p-values) ⩽ 0.05 threshold and were ranked by FDR values in an ascending order from the top to the bottom. The color bar is based on the *singscores'* values. **(A)** *singscores* calculated by the "No stable gene" method. Based on the top 3 clusters, hierarchal clustering correctly classified 73.4% (69/94) responders (left and middle clusters) and 54.9% (39/71) non-respond (right cluster). **(B)** *singscores* calculated by the "Skewed ranks" method. Based on the top 3 clusters, hierarchal clustering correctly classified 71.3% (67/94) responders (left and middle clusters) and 63.4% (45/71) non-respond (right cluster). **(C)** *singscores* calculated by the "HK genes" method. Based on the top 2 clusters, hierarchal clustering correctly classified 51.1% (48/94) responders (right cluster) and 76.1% (54/71) non-respond (left cluster).

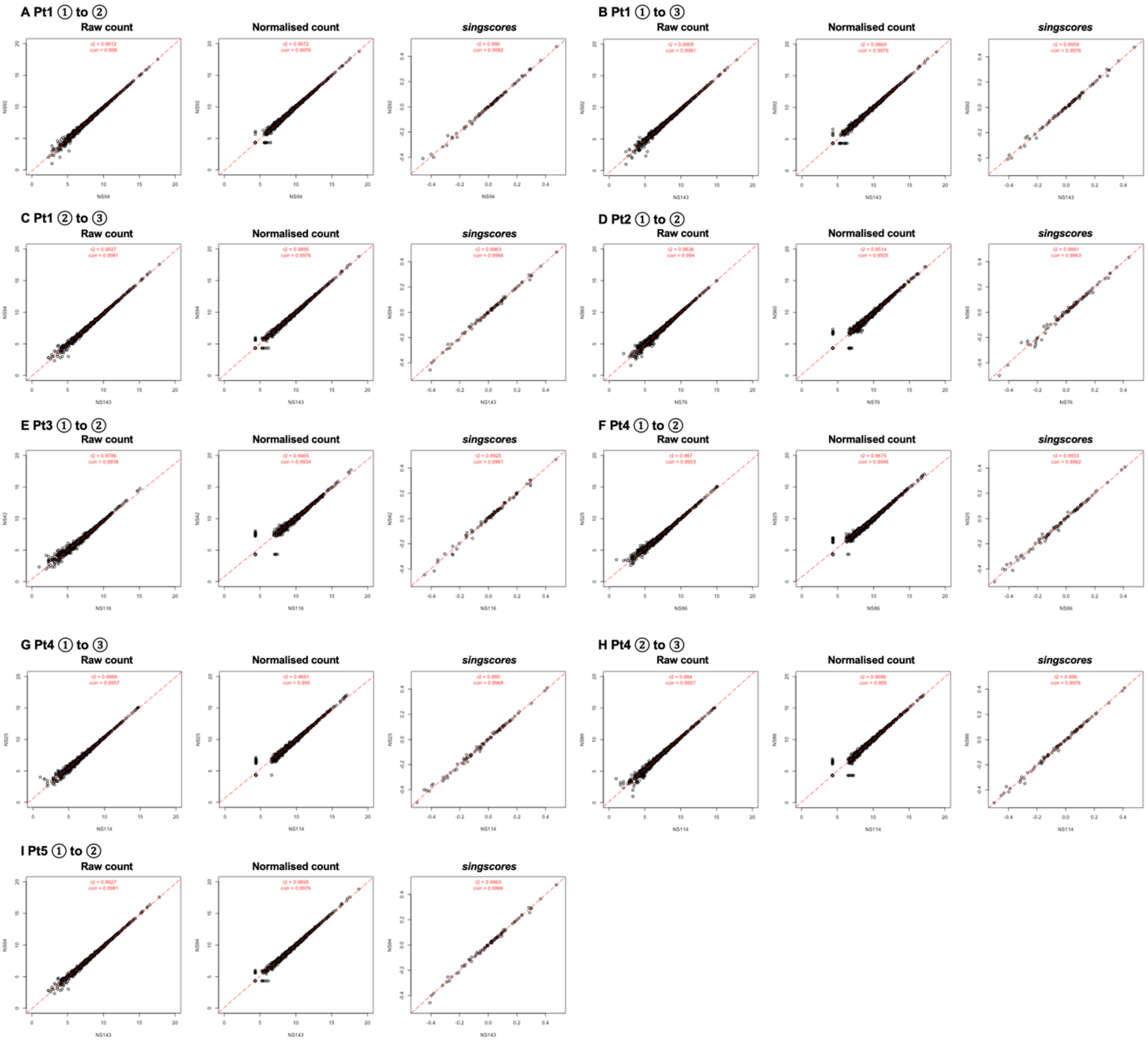

Figure S5. Linear regression plots among repeated samples. Each sub-plot has the Patient ID number which can map to Fig. 3A. Right plot: raw counts. Middle plot: normalised counts. Left plot: *singscores* of 81 signatures calculated from the "No stable gene" method. The diagonal red dashed line is the regression line when the *singscores* are identical between repeats.

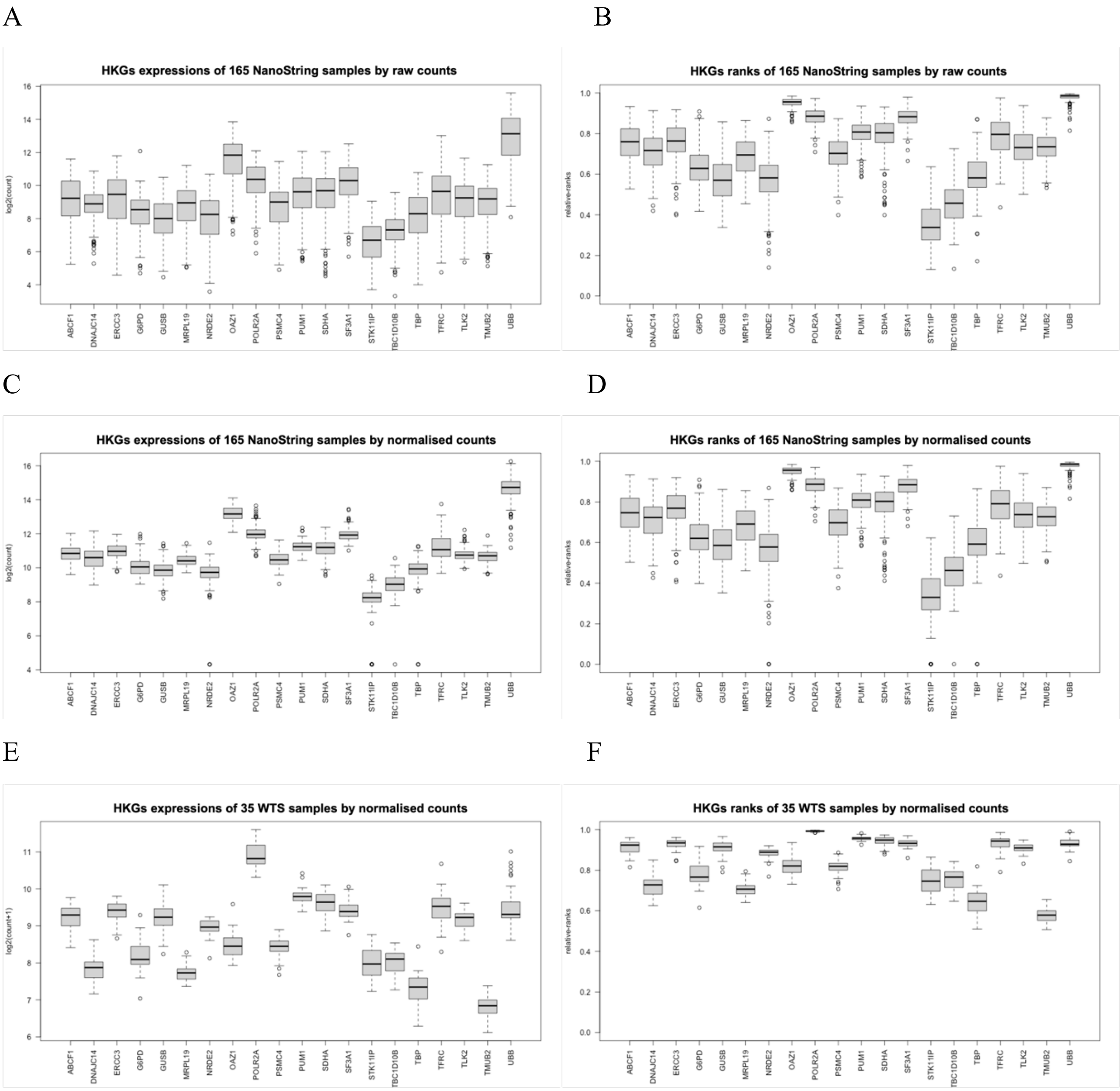

Figure S6. 20 Housekeeping gene expressions and ranks on NanoString and WTS platforms. (A, C, E) Boxplots of 20 housekeeping gene expressions in log_2-transformed count. **(B, D, F)** Boxplots of 20 housekeeping gene expression-relative ranks. The gene ranks were standardised by 770 in NanoString and by 22297 in WTS. **(A, B)** NanoString raw counts. **(C, D)** NanoString normalised counts. **(E, F)** WTS normalised counts.

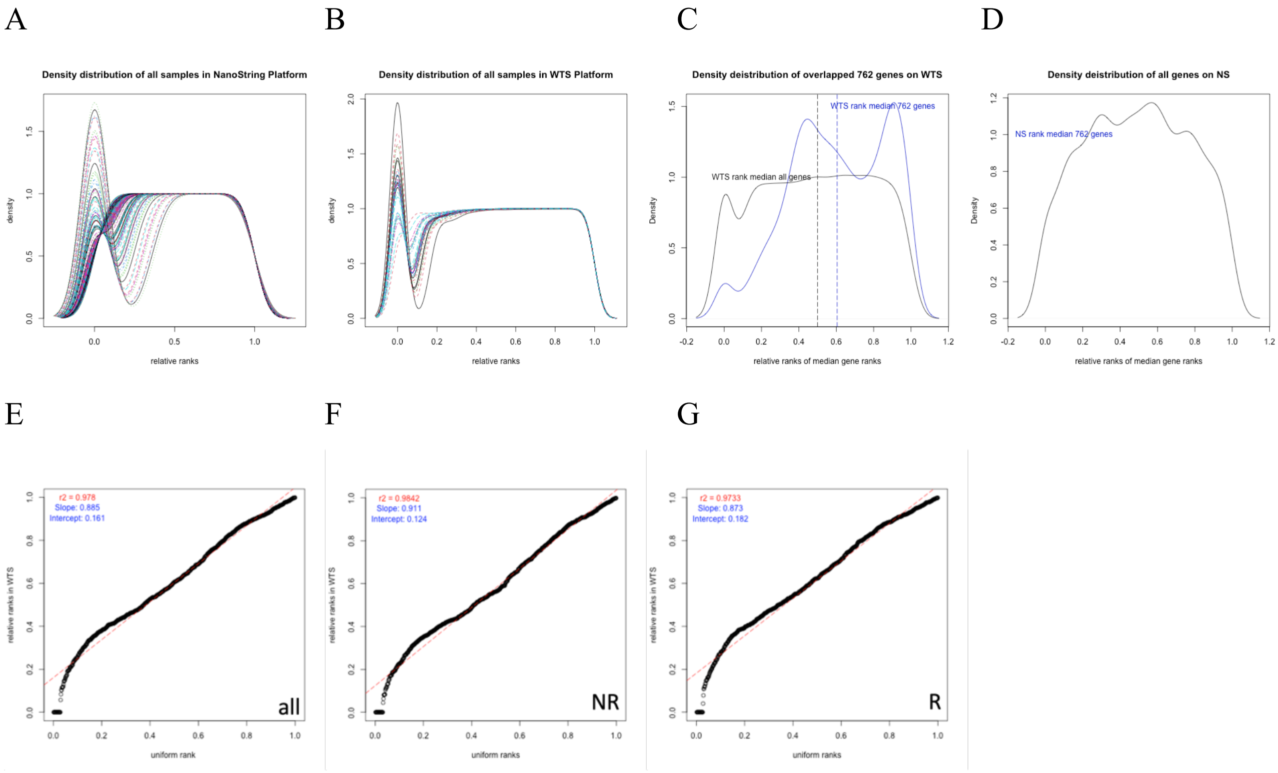

Figure S7. rank distributions. (A, B) Density plot on ranks per sample. **(A)** 165 NanoString samples with relative ranks (standardised by 770). **(B)** 35 WTS samples relative ranks (standardised by 22297). **(C, D)** Density plot on median ranks. **(C)** The black curve shows the density of relative median ranks (standardised by 22297) in all 22297 genes in WTS platform. The vertical black dashed line is the median of relative median ranks among 22297 genes. The blue curve shows the density of relative median ranks (standardised by 22297) in overlapping 762 genes in WTS platform. The vertical blue dashed line is the median of relative median ranks among 762 genes. **(D)** The curve shows the density of relative median ranks (standardised by 770) in overlapping 762 genes in NanoString (NS) platform. **(E-G)** Linear regression of median gene ranks in overlapping 762 genes from WTS platform against a uniform distributed rank 1:762. **(E)** using all 35 samples. **(F)** using non-responders only. **(G)** using responders only.

A

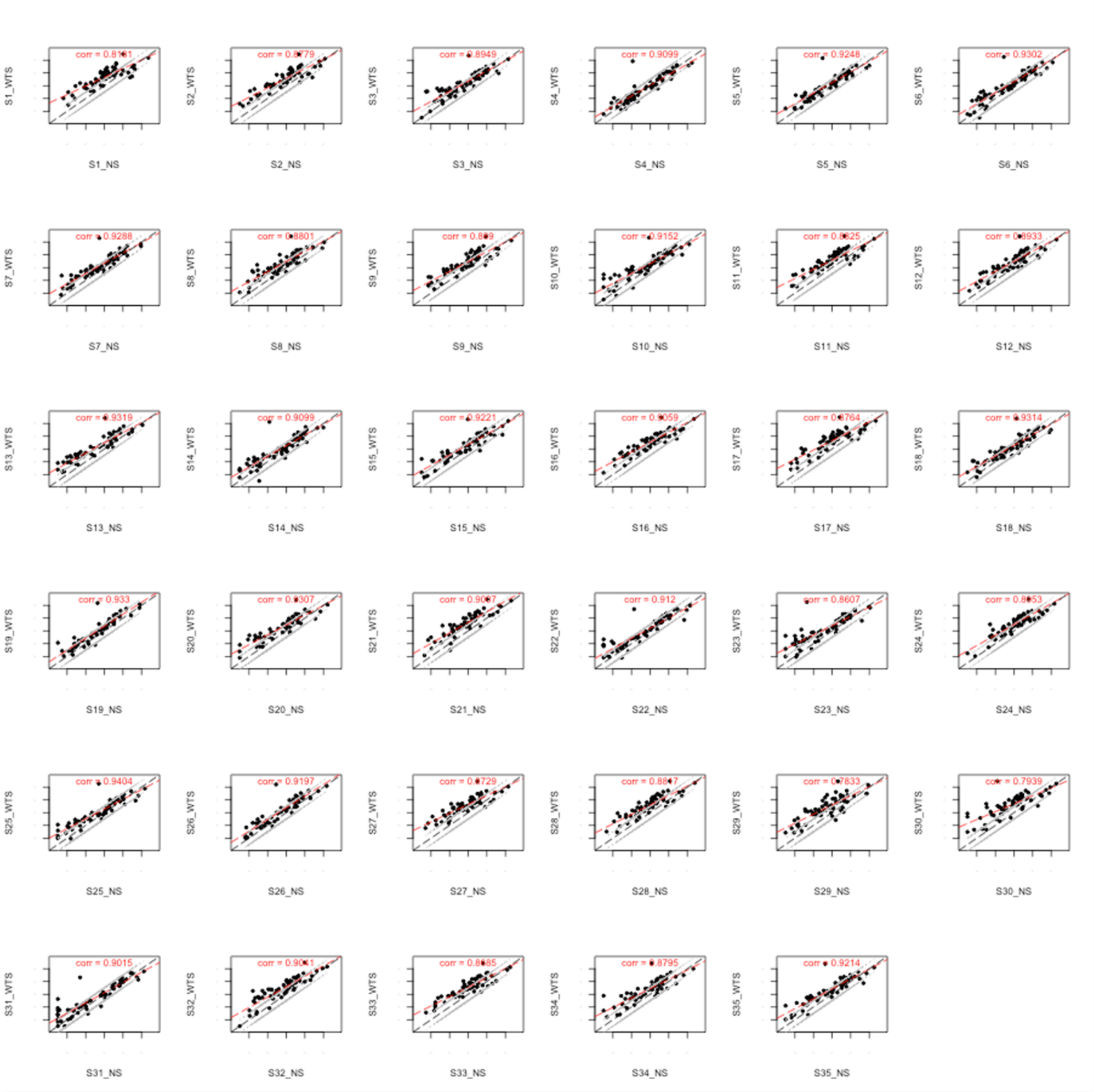

NS to WTS all

B

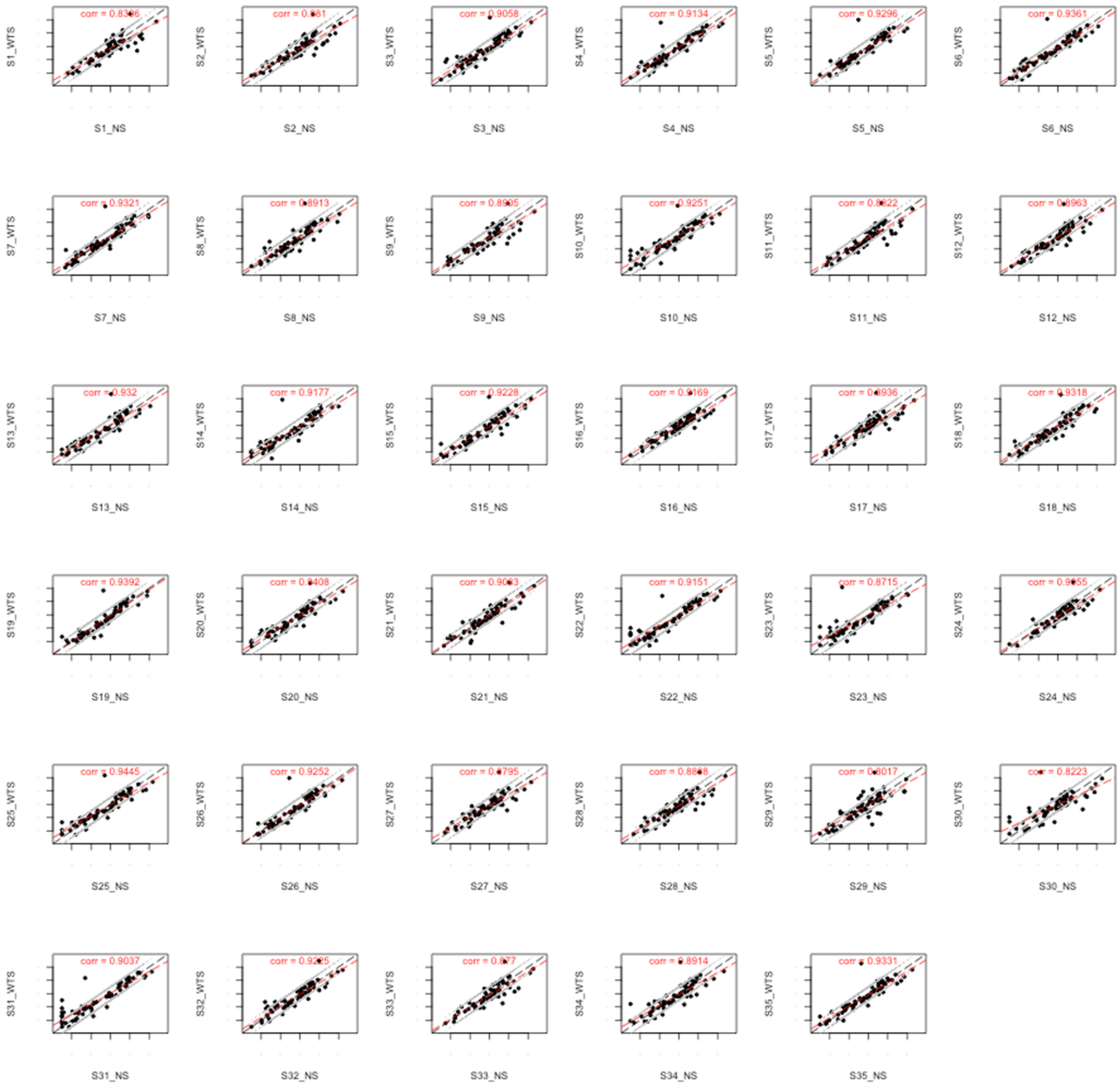

NS to WTS part

C

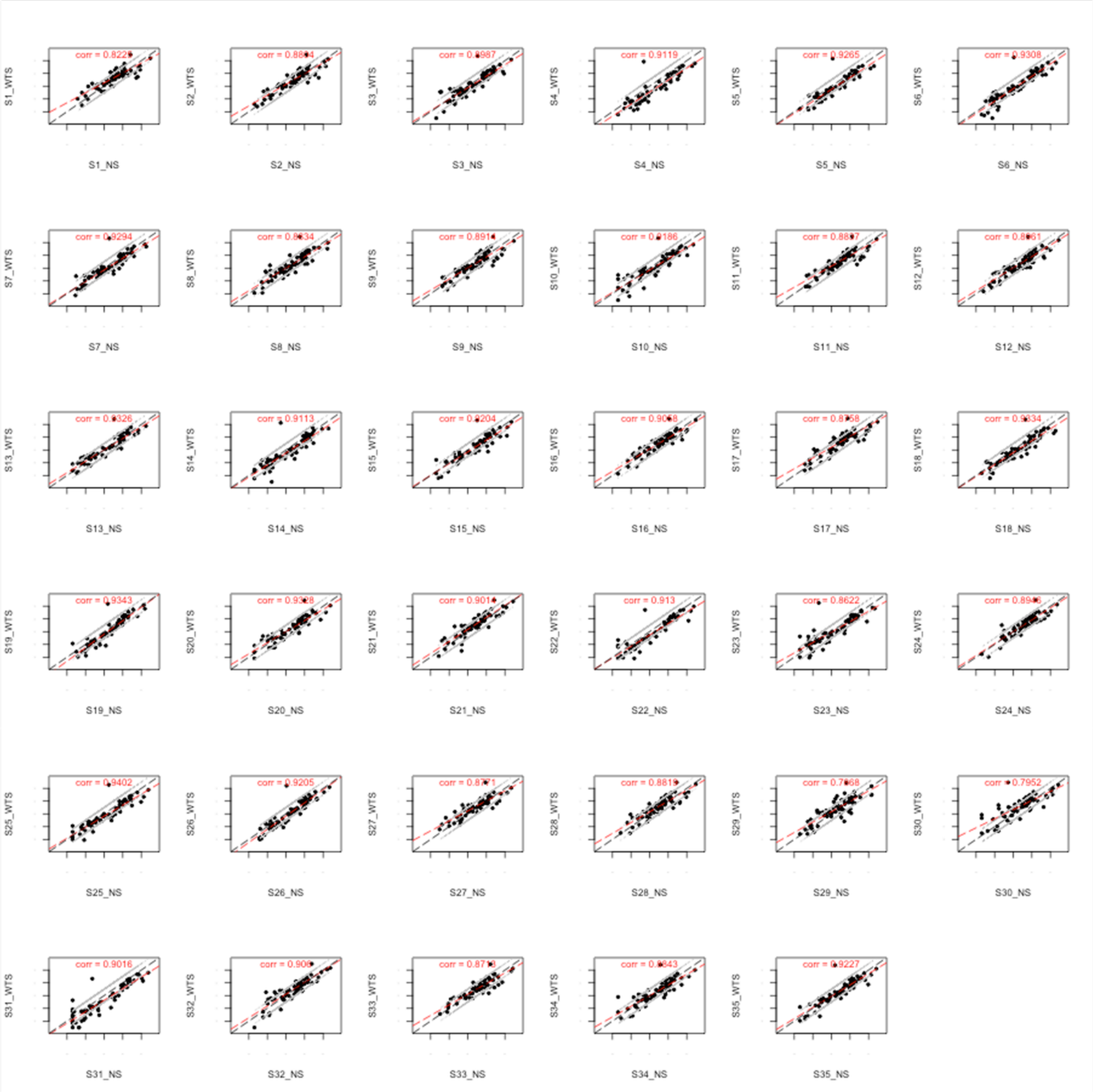

NS Skewed to WTS all

D

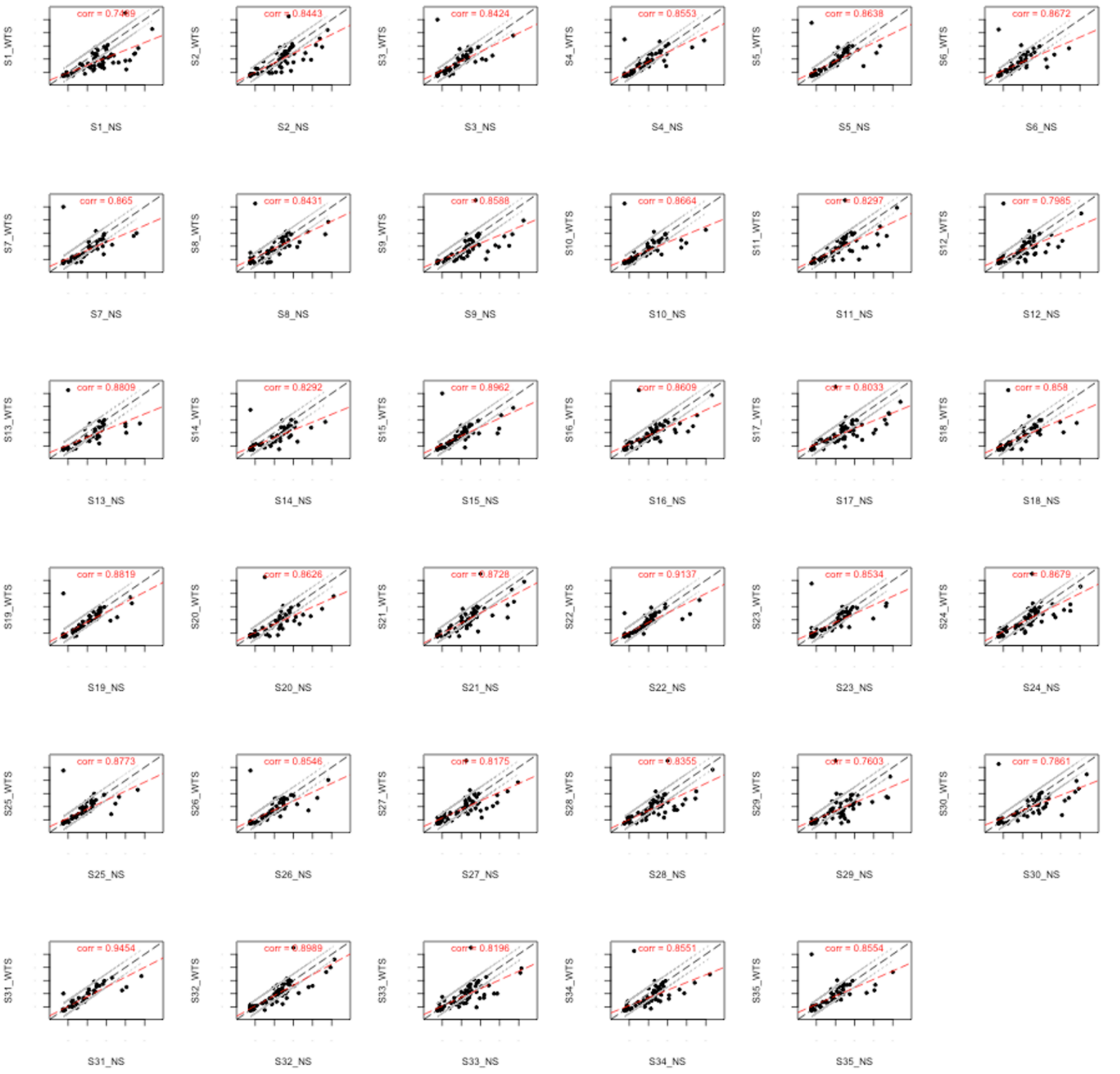
S1_WTS
corr = 0.7489
S1_NS
S2_WTS
corr = 0.8443
S2_NS
S3_WTS
corr = 0.8424
S3_NS
S4_WTS
corr = 0.8553
S4_NS
S5_WTS
corr = 0.8638
S5_NS
S6_WTS
corr = 0.8672
S6_NS
S7_WTS
corr = 0.865
S7_NS
S8_WTS
corr = 0.8431
S8_NS
S9_WTS
corr = 0.8588
S9_NS
S10_WTS
corr = 0.8664
S10_NS
S11_WTS
corr = 0.8297
S11_NS
S12_WTS
corr = 0.7985
S12_NS
S13_WTS
corr = 0.8809
S13_NS
S14_WTS
corr = 0.8292
S14_NS
S15_WTS
corr = 0.8962
S15_NS
S16_WTS
corr = 0.8609
S16_NS
S17_WTS
corr = 0.8033
S17_NS
S18_WTS
corr = 0.858
S18_NS
S19_WTS
corr = 0.8819
S19_NS
S20_WTS
corr = 0.8626
S20_NS
S21_WTS
corr = 0.8728
S21_NS
S22_WTS
corr = 0.9137
S22_NS
S23_WTS
corr = 0.8534
S23_NS
S24_WTS
corr = 0.8679
S24_NS
S25_WTS
corr = 0.8773
S25_NS
S26_WTS
corr = 0.8546
S26_NS
S27_WTS
corr = 0.8175
S27_NS
S28_WTS
corr = 0.8355
S28_NS
S29_WTS
corr = 0.7603
S29_NS
S30_WTS
corr = 0.7861
S30_NS
S31_WTS
corr = 0.9454
S31_NS
S32_WTS
corr = 0.8989
S32_NS
S33_WTS
corr = 0.8196
S33_NS
S34_WTS
corr = 0.8551
S34_NS
S35_WTS
corr = 0.8554
S35_NS

NS to WTS HK

E

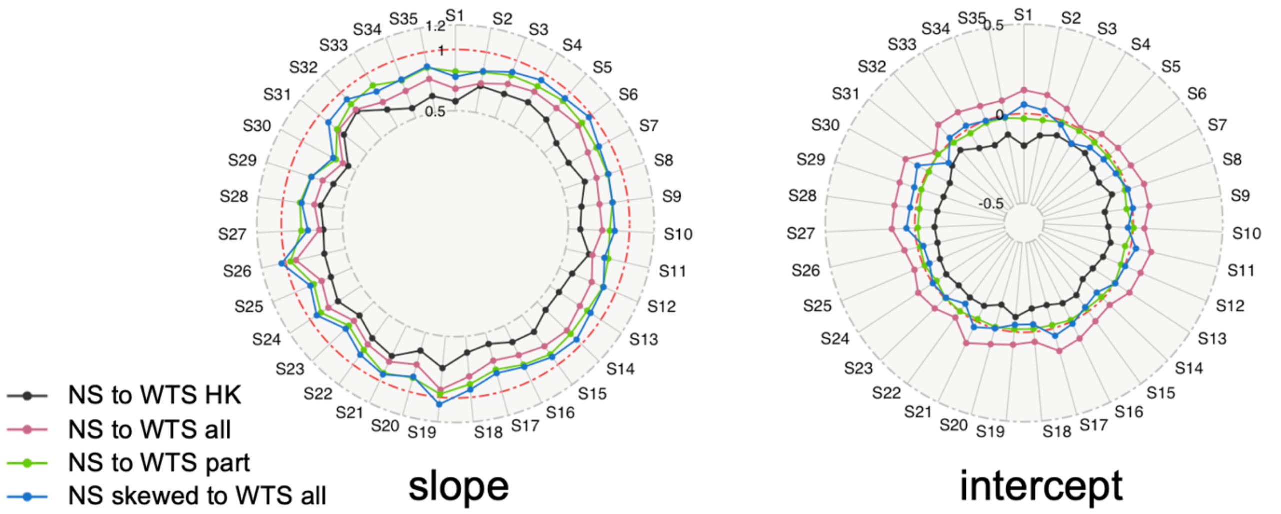

Figure S8. Linear regression plots for 35 overlapping samples based on all 63 overlapping signatures. Each dot is a signature's *singscore*. The x-axis is *singscores* from NanoString platform and y-axis is *singscores* from WTS platform. The red dotting line is the fitted linear regression line. The diagonal black dotted line represents the *singscores* from two platforms are identical which has r^2 = slope = 1 and intercept = 0. The signature locating in the area between two grey lines is considered as "consistent *singsocres*", which means absolute cross-platform difference in the signature is smaller than 0.1. **(A)** between the "No stable gene" method on NanoString to the "all" method on WTS. **(B)** between the "No stable gene" method on NanoString to the "part" method on WTS. **(C)** between the "Skewed ranks" method on NanoString to the "all" method on WTS. **(D)** between the "HK genes" method on NanoString to the "HK genes" method on WTS. **(E)** Radar plots of linear regression coefficients (slope and intercept) of 35 overlapping samples (S1-35). The format of plots is same to Fig. 5A.

A

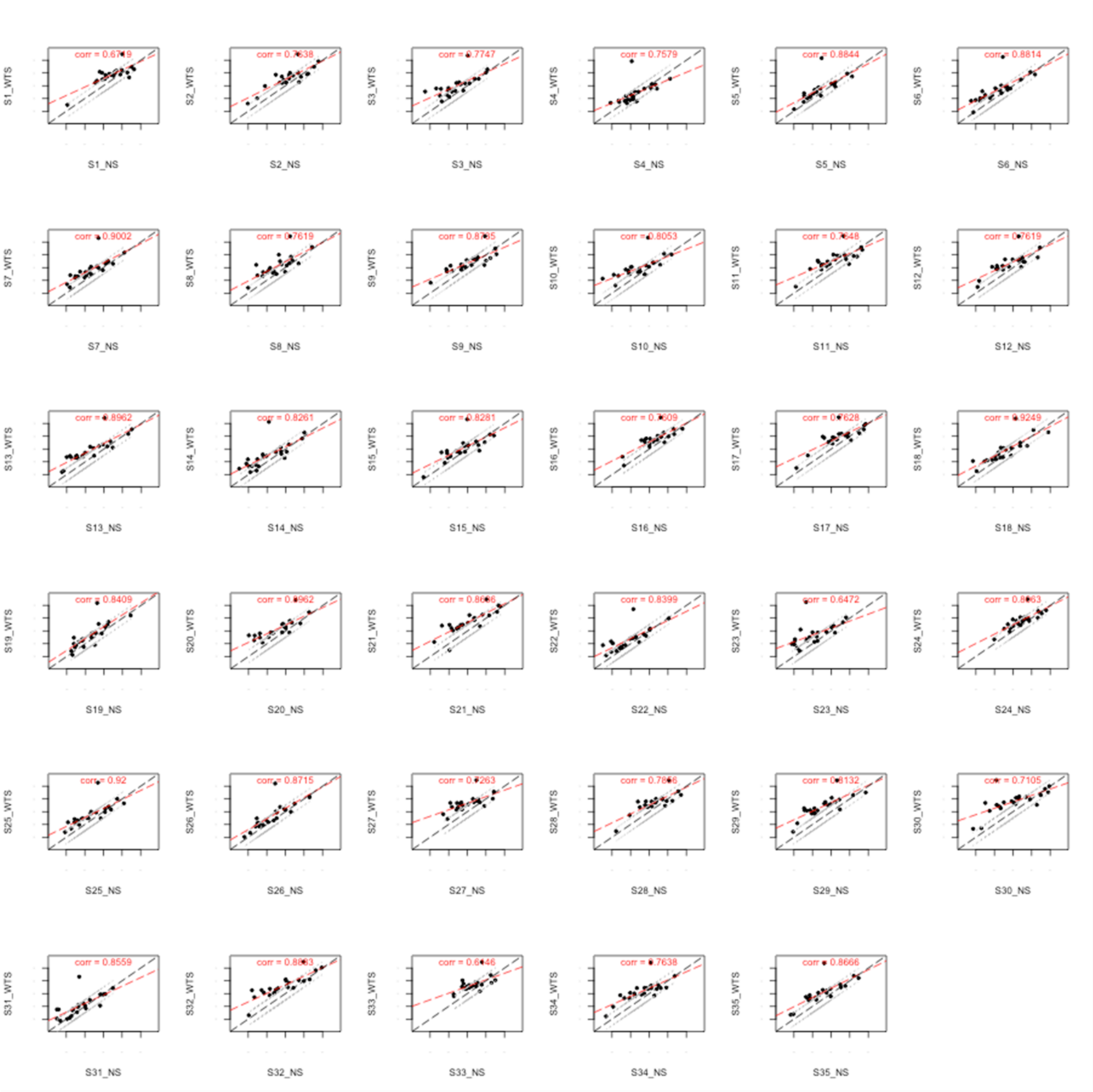

NS to WTS all

B

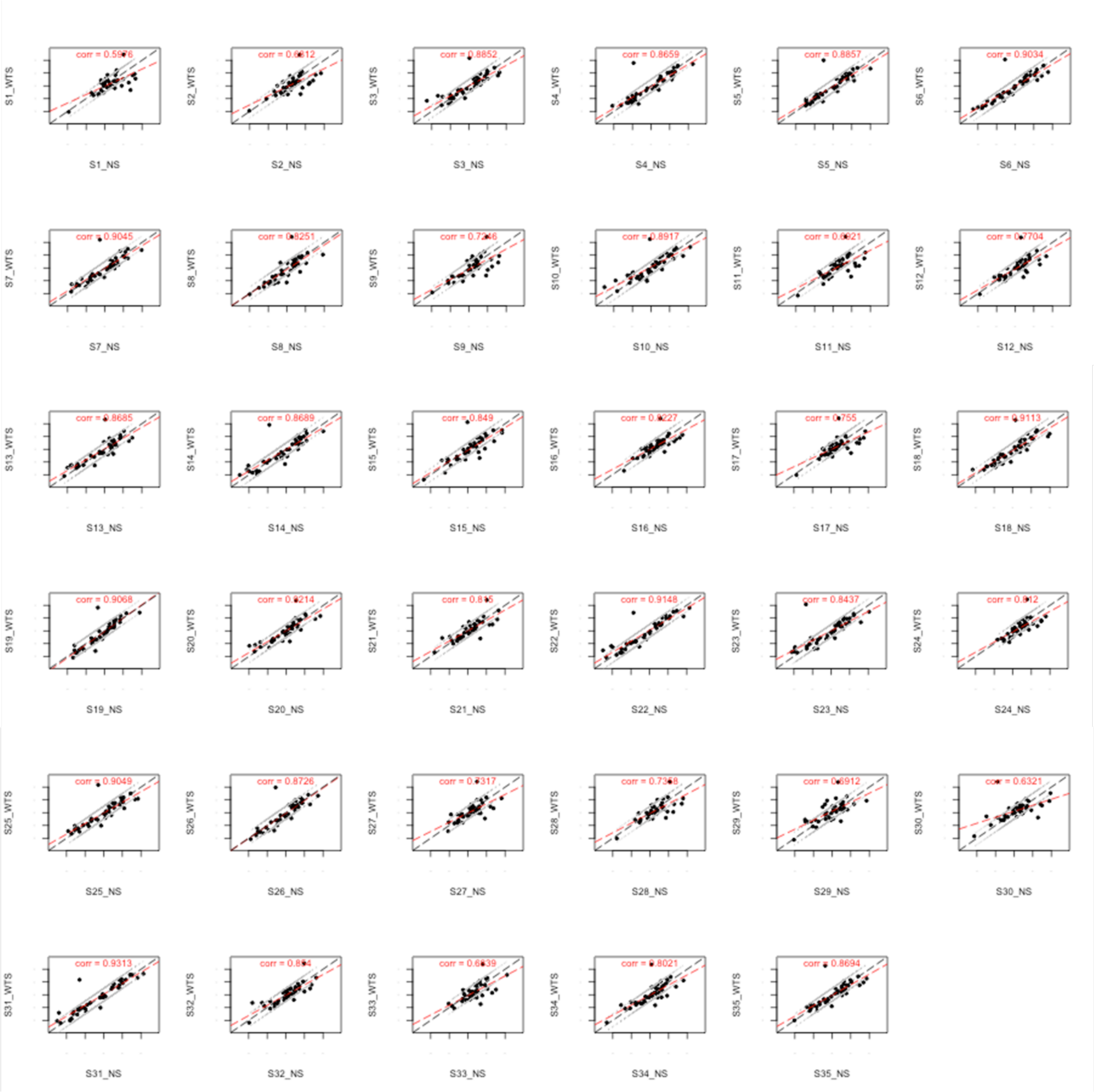

NS to WTS part

C

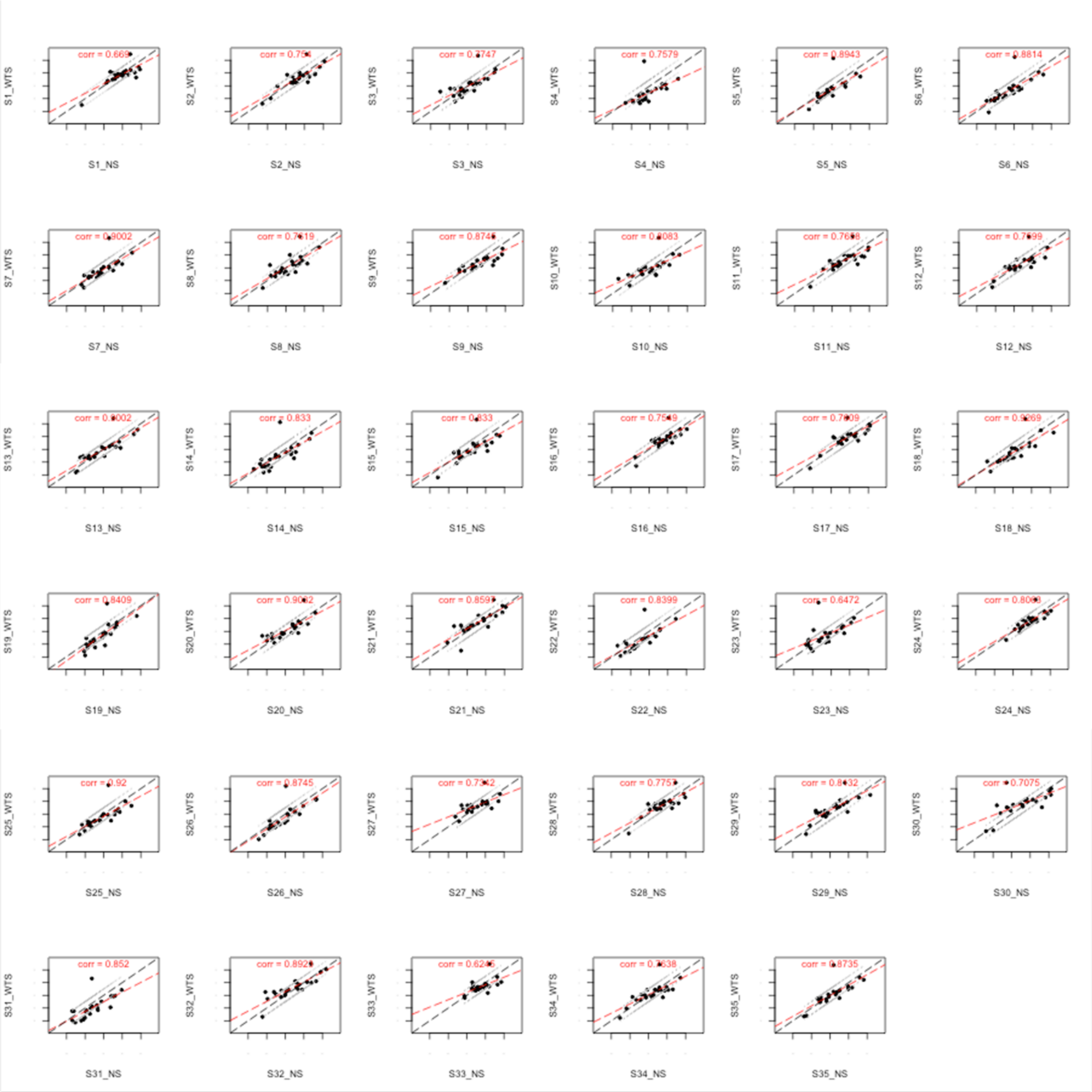

NS Skewed to WTS all

D

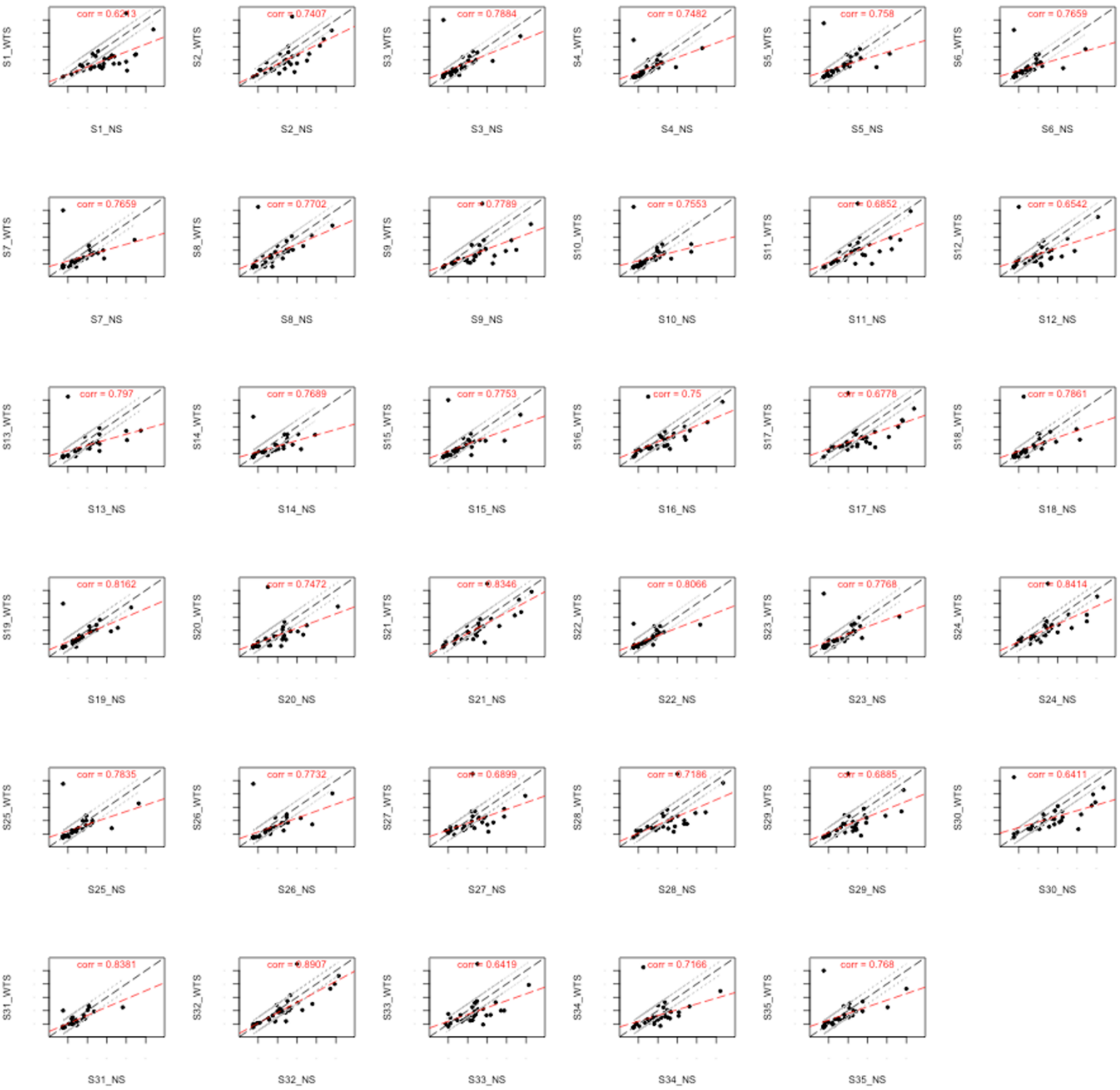
corr = 0.6213
S1_WTS
S1_NS
corr = 0.7407
S2_WTS
S2_NS
corr = 0.7884
S3_WTS
S3_NS
corr = 0.7482
S4_WTS
S4_NS
corr = 0.758
S5_WTS
S5_NS
corr = 0.7659
S6_WTS
S6_NS
corr = 0.7659
S7_WTS
S7_NS
corr = 0.7702
S8_WTS
S8_NS
corr = 0.7789
S9_WTS
S9_NS
corr = 0.7553
S10_WTS
S10_NS
corr = 0.6852
S11_WTS
S11_NS
corr = 0.6542
S12_WTS
S12_NS
corr = 0.797
S13_WTS
S13_NS
corr = 0.7689
S14_WTS
S14_NS
corr = 0.7753
S15_WTS
S15_NS
corr = 0.75
S16_WTS
S16_NS
corr = 0.6778
S17_WTS
S17_NS
corr = 0.7861
S18_WTS
S18_NS
corr = 0.8162
S19_WTS
S19_NS
corr = 0.7472
S20_WTS
S20_NS
corr = 0.8346
S21_WTS
S21_NS
corr = 0.8066
S22_WTS
S22_NS
corr = 0.7768
S23_WTS
S23_NS
corr = 0.8414
S24_WTS
S24_NS
corr = 0.7835
S25_WTS
S25_NS
corr = 0.7732
S26_WTS
S26_NS
corr = 0.6899
S27_WTS
S27_NS
corr = 0.7186
S28_WTS
S28_NS
corr = 0.6885
S29_WTS
S29_NS
corr = 0.6411
S30_WTS
S30_NS
corr = 0.8381
S31_WTS
S31_NS
corr = 0.8907
S32_WTS
S32_NS
corr = 0.6419
S33_WTS
S33_NS
corr = 0.7166
S34_WTS
S34_NS
corr = 0.768
S35_WTS
S35_NS

NS to WTS HK

E

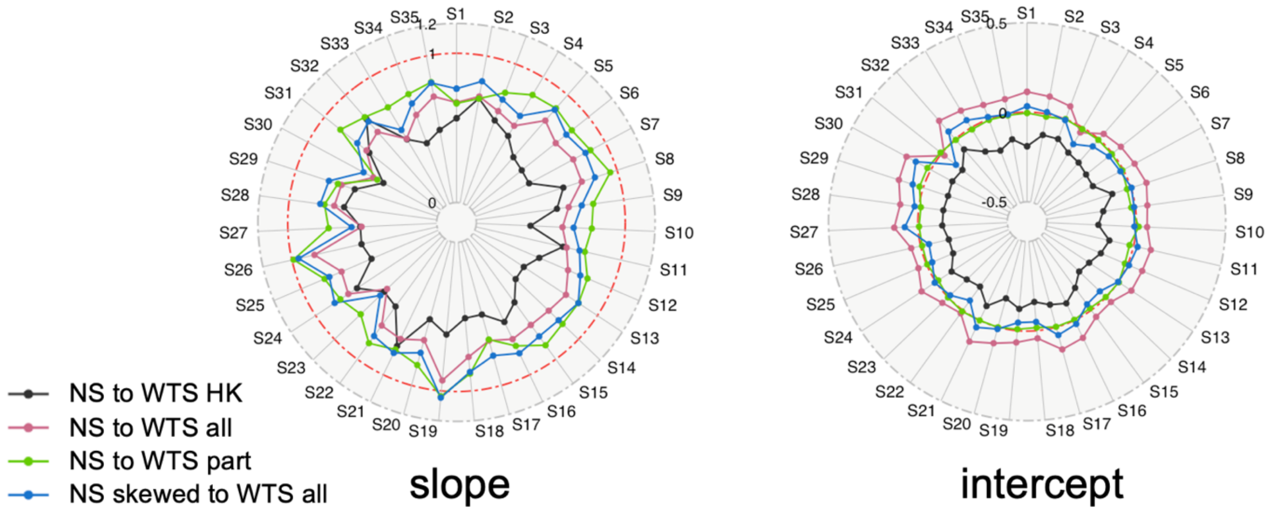

Figure S9. Linear regression plots for 35 overlapping samples based on highly correlated signatures only. Similar to the Fig. S8, but only the signatures with $r \geq 0.8$ were selected in each comparison. **(A)** between the "No stable gene" method in NanoString to the "all" method in WTS with 23 signatures included. **(B)** between the "No stable gene" method in NanoString to the "part" method in WTS with 40 signatures included. **(C)** between the "Skewed ranks" method in NanoString to the "all" method in WTS with 23 signatures included. **(D)** between the "HK genes" method in NanoString to the "HK genes" with 31 signatures included. **(E)** Radar plots of linear regression coefficients (slope and intercept) of 35 overlapping samples (S1-35). The format of plots is same to Fig. 5B.

A B

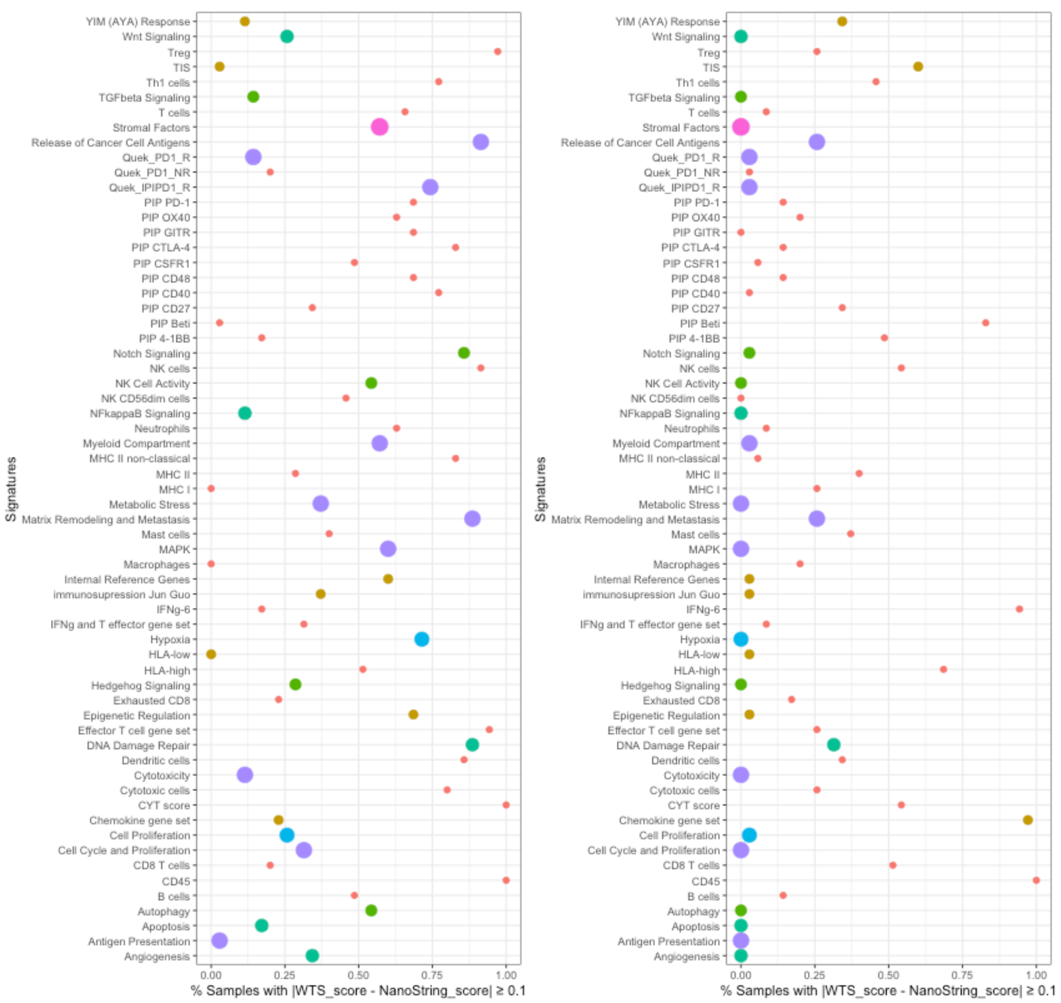
Signatures
% Samples with |WTS_score - NanoString_score| ≥ 0.1
YIM (AYA) Response
Wnt Signaling
Treg
TIS
Th1 cells
TGFbeta Signaling
T cells
Stromal Factors
Release of Cancer Cell Antigens
Quek_PD1_R
Quek_PD1_NR
Quek_IPIPD1_R
PIP PD-1
PIP OX40
PIP GITR
PIP CTLA-4
PIP CSFR1
PIP CD48
PIP CD40
PIP CD27
PIP Beti
PIP 4-1BB
Notch Signaling
NK cells
NK Cell Activity
NK CD56dim cells
NFkappaB Signaling
Neutrophils
Myeloid Compartment
MHC II non-classical
MHC II
MHC I
Metabolic Stress
Matrix Remodeling and Metastasis
Mast cells
MAPK
Macrophages
Internal Reference Genes
immunosupression Jun Guo
IFNg-6
IFNg and T effector gene set
Hypoxia
HLA-low
HLA-high
Hedgehog Signaling
Exhausted CD8
Epigenetic Regulation
Effector T cell gene set
DNA Damage Repair
Dendritic cells
Cytotoxicity
Cytotoxic cells
CYT score
Chemokine gene set
Cell Proliferation
Cell Cycle and Proliferation
CD8 T cells
CD45
B cells
Autophagy
Apoptosis
Antigen Presentation
Angiogenesis
0.00
0.25
0.50
0.75
1.00

C

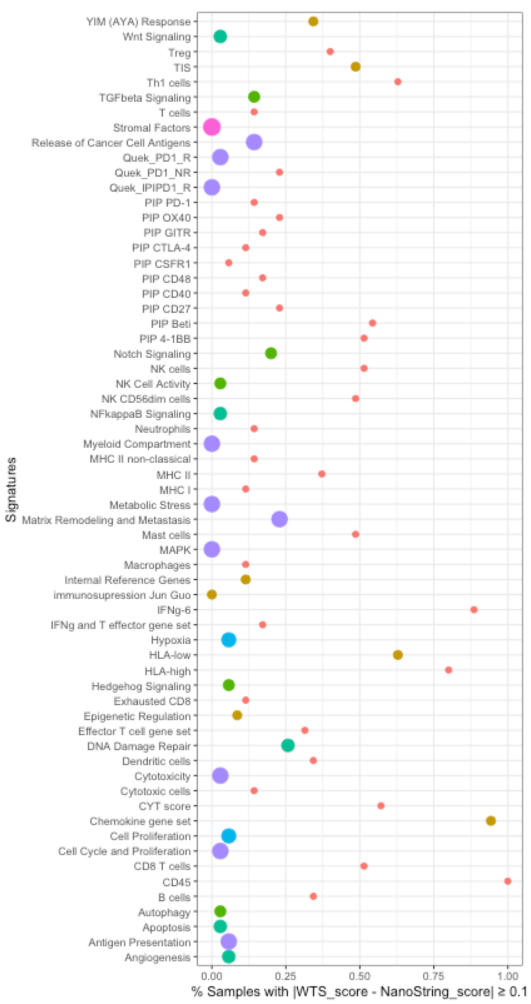

D

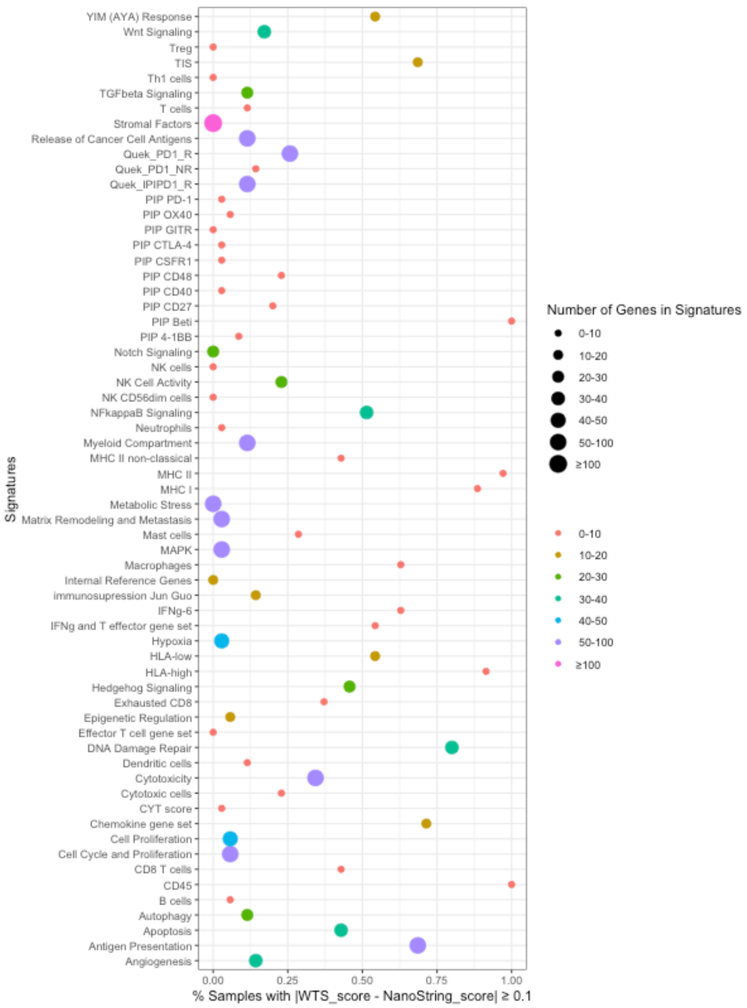

Figure S10. Cross-platform *singscores* consistency from the signature level. The x-axis is the percentage of overlapping samples displays absolute cross-platform *singscores* difference ≥ 0.1. **(A)** between the "No stable gene" method in NanoString to the "all" method in WTS. **(B)** between the "No stable gene" method in NanoString to the "part" method in WTS. **(C)** between the "Skewed ranks" method in NanoString to the "all" method in WTS. **(D)** between the "HK genes" method in NanoString to the "HK genes" in WTS.

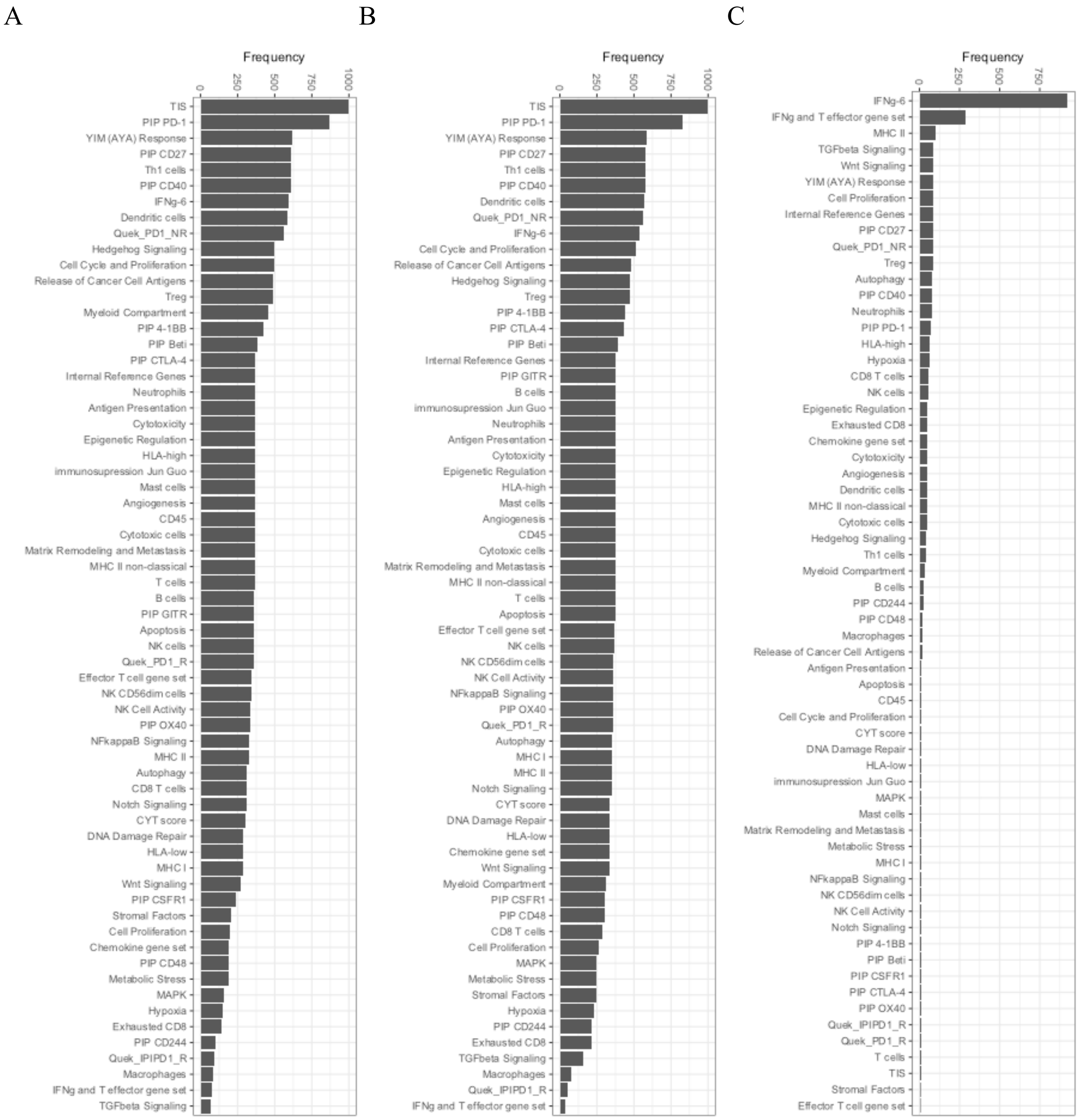

Figure S11. Frequency of non-zero coefficients. These bar plots show the frequency of each non-zero coefficients in 1000 times LASSO regressions. In each repeat, the non-zero coefficients were recorded under the λ value that provided the highest mean AUC in 10-fold CV LASSO regression. The 126 training samples used *singsocres* from the **(A)** “No stable gene” method, **(B)** “Skewed ranks” method, and **(C)** “HK genes” method.
